# Early threat experiences relate to reduced neural face discrimination in youth with emerging psychiatric symptoms: a frequency-tagging electroencephalography study

**DOI:** 10.1093/scan/nsaf105

**Published:** 2025-10-14

**Authors:** Zhiling Qiao, Celine Samaey, Stephanie Van der Donck, Victor Mazereel, Lise Jennen, Davy Vancampfort, Ruud van Winkel, Bart Boets

**Affiliations:** Faculty of Psychology, Shandong Normal University, Ji’nan, China; Shandong Provincial Key Laboratory of Brain Science and Mental Health, Shandong Normal University, Ji’nan, China; Center for Clinical Psychiatry, Department of Neurosciences, Research Group Psychiatry, KU Leuven, Leuven, Belgium; Center for Clinical Psychiatry, Department of Neurosciences, Research Group Psychiatry, KU Leuven, Leuven, Belgium; Center for Developmental Psychiatry, Department of Neurosciences, Research Group Psychiatry, KU Leuven, Leuven, Belgium; Center for Clinical Psychiatry, Department of Neurosciences, Research Group Psychiatry, KU Leuven, Leuven, Belgium; Center for Clinical Psychiatry, Department of Neurosciences, Research Group Psychiatry, KU Leuven, Leuven, Belgium; Department of Rehabilitation Sciences, KU Leuven, Leuven, Belgium; University Psychiatric Center (UPC), KU Leuven, Leuven, Belgium; Center for Clinical Psychiatry, Department of Neurosciences, Research Group Psychiatry, KU Leuven, Leuven, Belgium; University Psychiatric Center (UPC), KU Leuven, Leuven, Belgium; Leuven Brain Institute (LBI), KU Leuven, Leuven, Belgium; Child and Youth Institute, KU Leuven, Leuven, Belgium; Center for Developmental Psychiatry, Department of Neurosciences, Research Group Psychiatry, KU Leuven, Leuven, Belgium; Leuven Brain Institute (LBI), KU Leuven, Leuven, Belgium; Child and Youth Institute, KU Leuven, Leuven, Belgium

**Keywords:** childhood adversity, threat experiences, psychopathology, facial emotion processing, frequency-tagging EEG

## Abstract

Studies linking childhood adversity with risk for psychopathology suggest a threat-related information processing bias in those exposed. We combined frequency-tagging electroencephalography (EEG) and eye-tracking to assess automatic and implicit facial expression processing in youth aged 16–24 years with childhood adversity and emerging psychiatric symptoms (*N* = 52) as compared to healthy controls (*N* = 47). Neural discrimination of angry or happy faces from neutral faces was assessed via an EEG oddball paradigm. Neural responses and preferential looking towards angry versus neutral faces were quantified via an EEG multi-input paradigm with eye-tracking. Youth exposed to adversity showed reduced angry-neutral discrimination, which was specifically related to their threat but not neglect experiences and independent of concurrent psychiatric symptoms. When presenting angry and neutral faces simultaneously, controls showed higher neural responses to neutral faces but adversity exposed youth showed indistinct neural responses to both face categories. Furthermore, they showed increased neural responses for angry faces relative to controls. These results underscore the evidence of increased neural responses to angry faces in adversity as well as reduced neural threat-safety discrimination uniquely relating to threat experiences.

## Introduction

Childhood adversity is associated with the development of virtually all common forms of psychopathology (Baldwin et al. 2023), with evidence pointing towards comorbidity of anxiety, affective dysregulation and psychosis (Van Nierop et al. 2015). One of the postulated underlying factors is a threat-related information processing bias (Mclaughlin and Lambert 2017), in particular with regard to facial emotional cues, which we heavily rely on to communicate emotional states. Thus far, however, the evidence on facial expression processing in relation to childhood adversity does not consistently converge (da Silva Ferreira et al. 2014, Doretto and Scivoletto 2018, Medeiros et al. 2020, Saarinen et al. 2021, Annie et al. 2023). Three critical factors might contribute to this mixed evidence. First, the dimensional framework of childhood adversity suggests that experiences of threat (e.g. domestic violence, physical and sexual abuse, and other types of interpersonal violence) versus deprivation (e.g. institutionalization, neglect, poverty) may differentially impact on emotional development (McLaughlin et al. 2014, McLaughlin and Sheridan 2016). Yet, previous studies either mainly incorporated only one dimension or they mixed both dimensions of adverse experiences, thereby impeding the disentanglement of distinct and unique effects (but see Pollak et al. 2000, Nelson et al. 2013, Schäfer et al. 2023).

Second, a range of psychiatric disorders, such as schizophrenia-spectrum disorders, bipolar disorder and depression, are also characterized by deficient facial expression processing (Catalan et al. 2016, Cotter et al. 2018, Pena-garijo et al. 2023). While the association between childhood adversity and psychopathology is well established, the exact status and role of altered facial expression processing in adversity victims who also present psychiatric symptoms is still unclear (Samaey et al. 2020), and limited studies have explored this explicitly (Shackman et al. 2007, Briggs-Gowan et al. 2015, Aas et al. 2017, Bodenschatz et al. 2019, Peters et al. 2019, Kirkham and Levita 2020, Saarinen et al. 2021).

Third, most previous studies utilized explicit behavioural emotion recognition paradigms, such as labelling facial expressions (Pollak and Tolley-Schell 2003, Humphreys et al. 2016, Bertó et al. 2017). This explicit judgment of facial expressions can be influenced by many confounding factors, such as motivation, task understanding, and compensatory strategies (Rutherford and McIntosh 2007, Harms et al. 2010). Moreover, even the more implicit eye-tracking paradigms aimed at capturing attentional orientation, may not necessarily capture the saliency and preferential processing of particular facial cues, as attention also comprises covert orienting, which does not necessarily converge with overt looking behaviour (Posner and Petersen 1990, Petersen and Posner 2012).

Against this background, the current study investigates facial expression processing in a youth group (aged 16–24 years) who experienced childhood adversity and who presents (sub)clinical symptoms of depression, anxiety and/or psychosis, and a control group. We utilize a state-of-the-art frequency-tagging electroencephalography (EEG) approach. This approach elicits brain responses exactly tagged to the presentation rate of stimuli and allows to objectively mark and quantify the automatic neural processing without explicit task demands (Liu-Shuang et al. 2014, Dzhelyova et al. 2017). In addition to its robustness, high signal-to-noise ratio and high test–retest reliability (Dzhelyova et al. 2019, Qiao et al. 2022, Van der Donck et al. 2022), it is particularly sensitive to pinpoint subtle individual differences in facial expression processing (Coll et al. 2019, Poncet et al. 2019, Van der Donck et al. 2019, 2020, Baudouin et al. 2023, Naumann et al. 2025). We administered an oddball paradigm to investigate facial expression discrimination, i.e. the neural ability to automatically discriminate angry and happy faces from neutral faces. Based on prior findings demonstrating enhanced identification of angry faces (Pollak and Sinha 2002), we expected to observe increased discrimination of angry faces in participants with adversity relative to controls. In addition, we administered a multi-input paradigm presenting angry and neutral faces simultaneously, to assess both the neural processing (via frequency-tagging EEG) and overt attentional orienting (via eye-tracking) towards each stimulus category. Given previous evidence of attentional bias towards anger (Pollak and Tolley-Schell 2003), we expected to observe selectively increased neural responses and preferential looking (i.e. attentional bias) towards angry faces in participants with adversity relative to controls.

## Methods

### Participants

Ninety-nine youths were recruited through posters and flyers distributed in leisure centres, schools, medical practices, health centres and specialized centres providing care for youth. Based on pre-defined cut-off scores of childhood adversity, and depression, anxiety and psychotic symptoms, two participant groups were included: a childhood adversity group (CA, *n* = 52), who scored above the threshold of adversity and presented substantial symptoms across at least two symptom dimensions, and a healthy control group (HC, *n* = 47) with participants who scored below all of the thresholds. Childhood adversity exposure (before 18 years of age) was measured using a modified screening version of the Juvenile Victimization Questionnaire 2nd revision (Finkelhor et al. 2005) and 5 questions of the Emotional Neglect subscale of the Childhood Trauma Questionnaire (Bernstein and Fink 1998). Current depressive, anxiety and psychotic symptoms were measured using the Beck Depression Inventory (Beck et al. 1996, Whisman and Richardson 2015), the State Trait Anxiety Inventory (Spielberger et al. 1983, Addolorato et al. 1999, Dennis et al. 2013) and the Prodromal Questionnaire-16 version (Savill et al. 2018), respectively. Full details of participant recruitment and measures of adversity and symptoms are reported in Supplementary Appendices S1 and S2. During the test session, adversity and symptoms were measured again to obtain more details on adversity exposure and to assess current symptomatology. Based on this concurrent assessment, individual scores for general adversity exposure, threat exposure, neglect exposure, and symptomatology were calculated (Supplementary Appendix S2).

### Stimuli and procedure

**Figure 1.**
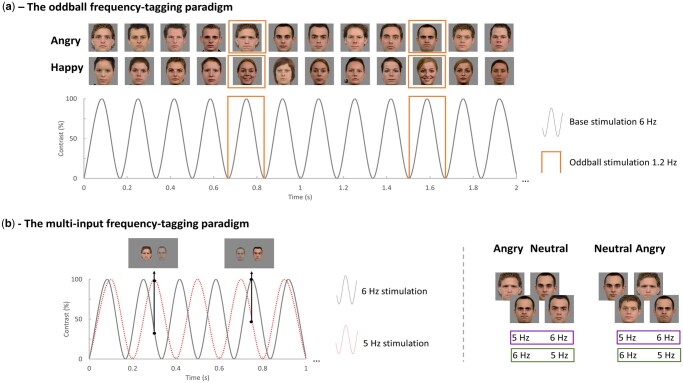
The frequency-tagging oddball paradigm and multi-input paradigm. (a) Illustration of a stimulation sequence for the oddball paradigm, with neutral faces presented at a 6 Hz base rate, periodically interleaved with either an angry or happy every fifth image (1.2 Hz oddball rate). In total, eight sequences (i.e. two male and two female sequences for each of the two expressions) were administered and the sequence order was randomized for each participant. (b) Left: Illustration of a stimulation sequence for the multi-input paradigm, with angry faces presented at 5 Hz in the left visual field and neutral faces presented at 6 Hz in the right visual field. The centre of each face image was 155 pixels away from the centre of the screen. In total, eight sequences were administered, with the order randomized for each participant. The first black arrow indicates that the angry face was presented at 100% and the neutral face was presented at around 59% contrast at 0.3 s. The second black arrow indicates that the angry face was presented at around 51% and the neutral face was presented at 100% at 0.75 s. Right: The presentation rates were counterbalanced.

Stimuli (Supplementary Appendix S3) for both paradigms consisted of facial images of 13 men and 13 women with angry, happy and neutral expressions, selected from the Radboud Faces Database (Langner et al. 2010). For the oddball paradigm (Fig. 1a), a series of base stimuli (i.e. neutral faces) were displayed at 6 Hz, periodically interleaved with either an angry or happy face (in different sequences) every fifth image (i.e. 6 Hz/5 = 1.2 Hz oddball rate). The multi-input paradigm (Fig. 1b) consisted of two simultaneously presented streams of neutral and angry faces, tagged with different presentation rates (5 Hz vs. 6 Hz or vice versa) and presented at either the left or right visual field. Sequences lasted 60 s for the oddball paradigm and 30 s for the multi-input paradigm, and were flanked by 2 s of fade-in and fade-out, with the stimuli contrast gradually increasing (0–100%) or decreasing (100–0%). Eight sequences were conducted for the oddball paradigm (4 angry and 4 happy) and eight for the multi-input paradigm. To guarantee a constant level of attention, an orthogonal task was implemented, asking participants to press a key as soon as they noticed a brief colour change of the fixation cross presented in the centre of the images (the oddball paradigm) or the appearance of a rectangle around the two images (the multi-input paradigm).

### Data analysis

**EEG data** was preprocessed using Letswave 6 (https://www.letswave.org/) and MATLAB 2021a (Supplementary Appendix S5). After preprocessing, data was transformed from the time domain to the frequency domain using a fast Fourier transformation, yielding amplitude spectra with spectral resolutions of 0.017 Hz and 0.033 Hz for the oddball and multi-input paradigm, respectively. The frequency domain data contain the responses tagged to the stimulation frequencies and their harmonics (i.e. integer multiples). To obtain a measure of neural responses to (differences in) facial expressions, we calculated baseline-subtracted amplitudes at the target frequencies and their harmonics by subtracting the average amplitude level of the 20 surrounding bins from the amplitude of the target frequency bin. Additionally, for visualization, we calculated signal-to-noise ratio (SNR) by dividing the amplitude of the frequency bin of interest by the average amplitude of the 20 surrounding frequency bins, as this allows to visualize even small response amplitudes with high SNR. To determine the number of harmonics to be included in analyses, we calculated Z-scores based on the mean and s.d. of these 20 surrounding frequency bins. Harmonics were included if the Z-score for two consecutive harmonics was above 1.64 (*P* < .05) in all relevant regions of interest (ROIs) for both groups and all conditions per paradigm. Consequently, the oddball discrimination responses were quantified as the summed responses of the first six harmonics (i.e. until 6F/5 = 7.2 Hz) without the harmonic corresponding to the 6 Hz base rate frequency. For the multi-input paradigm, the responses were quantified as the summed responses of the first four harmonics for both frequencies (i.e. until 20 Hz and 24 Hz for 5 Hz and 6 Hz, respectively).

Based on previous studies (Van der Donck et al. 2020, 2022) and supported by visual inspection of the topographical maps, we defined the following ROIs for the oddball paradigm: left occipito-temporal (LOT; P7, P9, PO7), right occipito-temporal (ROT; P8, P10, PO8) and medial occipital (MO; Iz, Oz, O1, O2) regions. The same MO region was also selected for the multi-input paradigm.

**Eye-tracking data** was analysed using a series of custom-built Matlab scripts (Supplementary Appendix S5). Two areas of interest (AOI) were defined as the rectangular areas where the two faces were presented. An additional ‘outside AOI’ was defined to label all the fixation points that were not attributed to the two AOIs. Proportional looking times for each AOI were then quantified as the duration of all fixation points allocated to that AOI using a probability weighting approach while taking the subject-specific data quality into account.

### Statistical analysis

Linear mixed models (LMM) were performed using R-package nlme, version 3.1-163 (Pinheiro et al. 2024), in RStudio, version 4.1.3 (R Core Team 2022). Tukey-corrected post-hoc t-tests were performed using R-package emmeans, version 1.7.3 (Lenth et al. 2022).

For both paradigms, the main analyses followed three steps: (i) group-level comparisons between healthy controls (HC) and individuals with CA, (ii) examination of the continuous effect of general adversity exposure within the CA group controlling for symptomatology, and (iii) assessment of the distinct continuous effect of threat and neglect exposure, controlling for each other and for symptomatology. Specifically, for the neural discriminative responses measured in the oddball paradigm, we constructed the following three linear mixed models (LMMs): Model_1_, y ∼ age + sex + ROI + Group * Expression + (1|subject), with Group [HC vs. CA] as the between-subjects effect and Expression [angry vs. happy] and ROI [LOT, MO, ROT] as the within-subjects effects; Model_2_, y ∼ age + sex + Childhood adversity * Expression + Depression * Expression + Anxiety * Expression + Psychosis * Expression + (1|subject), with adversity and symptoms as continuous measures; Model_3_, y ∼ age + sex + Threat * Expression + Neglect * Expression + Depression * Expression + Anxiety * Expression + Psychosis * Expression + (1|subject), with threat and neglect experiences and symptoms as continuous measures. Similar analysis steps were conducted for neural responses to the visual base stimulation (Supplementary Appendices S6 and S8). For the neural responses and looking patterns measured during the multi-input paradigm, three similar models were constructed, with Expression [angry vs. neutral] as the within-subjects effect. Dimensional measures for adversity and symptom levels were standardized (mean = 0, SD = 1). Due to the correlation between adversity and symptoms, multicollinearity was assessed by computing the variance inflation factor and results revealed that all values were lower than 5, indicating no problematic amount of collinearity (James et al. 2021).

To investigate the separate effect of adversity and symptoms (as dimensional measures), i.e. while not controlling for each other, exploratory analyses were also conducted (Supplementary Appendix S6) and the results support our main findings (Supplementary Appendices S8 and S9).

## Results

### Demographic information

The following participant samples were included in the final analyses: 47 HC and 46 CA for the oddball paradigm, 41 HC and 46 CA for the multi-input EEG data, and 28 HC and 39 CA for the eye-tracking data (Table 1). A summary of the participant enrolment is reported in Supplementary Appendix S7. Good test-retest reliability of adversity and symptomatology measures during screening and testing was confirmed and childhood adversity was effectively shown to be associated with depressive, anxiety and psychotic symptoms in the CA group (Supplementary Appendix S7).

**Table 1.** Demographic and clinical information during the test session of the final participant samples that were included for each paradigm.

Characteristics		The oddball paradigm			The multi-input paradigm			The multi-input paradigm		
		EEG			EEG			Eye tracking		
		HC	CA	*P* value	HC	CA	*P* value	HC	CA	*P* value
Sex, female/male		30/17	30/16	.829	28/13	30/16	.761	20/8	24/15	.400
Age, years: mean (s.d.)		21.3 (1.9)	20.0 (2.2)	.005	21.4 (1.9)	20.0 (2.2)	.004	21.5 (2.1)	20.2 (2.2)	.024
Childhood adversity, average score: mean (s.d.)	Adversity	0.03 (0.06)	0.54 (0.43)	<.001	0.03 (0.06)	0.54 (0.43)	<.001	0.03 (0.04)	0.52 (0.41)	<.001
	Threat	0.04 (0.07)	0.49 (0.39)	<.001	0.04 (0.07)	0.49 (0.39)	<.001	0.03 (0.04)	0.47 (0.35)	<.001
	Neglect	0.03 (0.11)	0.67 (0.78)	<.001	0.02 (0.09)	0.67 (0.78)	<.001	0.03 (0.11)	0.64 (0.76)	<.001
Symptoms, score: mean (s.d.)	Depression	2.85 (2.92)	19.70 (10.20)	<.001	2.93 (3.04)	19.70 (10.20)	<.001	2.34 (2.62)	19.2 (9.81)	<.001
	Anxiety	30.50 (5.27)	53.7 (10.10)	<.001	30.60 (5.37)	53.70 (10.10)	<.001	30.0 (4.77)	52.8 (9.61)	<.001
	Psychosis	1.11 (1.43)	5.20 (3.42)	<.001	1.12 (1.49)	5.20 (3.42)	<.001	1.07 (1.27)	5.26 (3.45)	<.001

Childhood adversity was calculated as a continuous score by averaging the mean frequency scores across seven categories (peer and sibling victimization/bullying, physical abuse, physical neglect, emotional abuse, emotional neglect, sexual abuse and domestic violence), assessed using a modified version of the Juvenile Victimization Questionnaire 2nd revision (Adult Retrospective Form; JVQ-R2) and the Emotional Neglect subscale of the Childhood Trauma Questionnaire (CTQ). We derived two composite measures: neglect exposure (mean of physical and emotional neglect scores) and threat exposure (mean of the remaining five categories). Psychiatric symptoms were assessed with the Beck Depression Inventory (BDI-II) for depression, the trait scale of the State Trait Anxiety Inventory for anxiety, and the symptom scale of the Prodromal Questionnaire-16 (PQ-16) for psychotic symptoms. Group comparisons were performed using independent samples *t*-tests for continuous variables and chi-square tests for sex distribution.

### Discrimination of angry versus neutral and happy versus neutral faces in the oddball paradigm

#### Base stimulation

No significant main or interaction effect for group was observed, indicating a similar synchronization to the flickering stimuli in both groups (see details in Supplementary Appendix S8). Likewise, dimensional analyses within the CA group revealed no significant associations with childhood adversity (general adversity exposure, threat, and neglect) and psychiatric symptoms (depression, anxiety, and psychosis) (Supplementary Appendix S8).

#### Oddball responses

**Figure 2.**
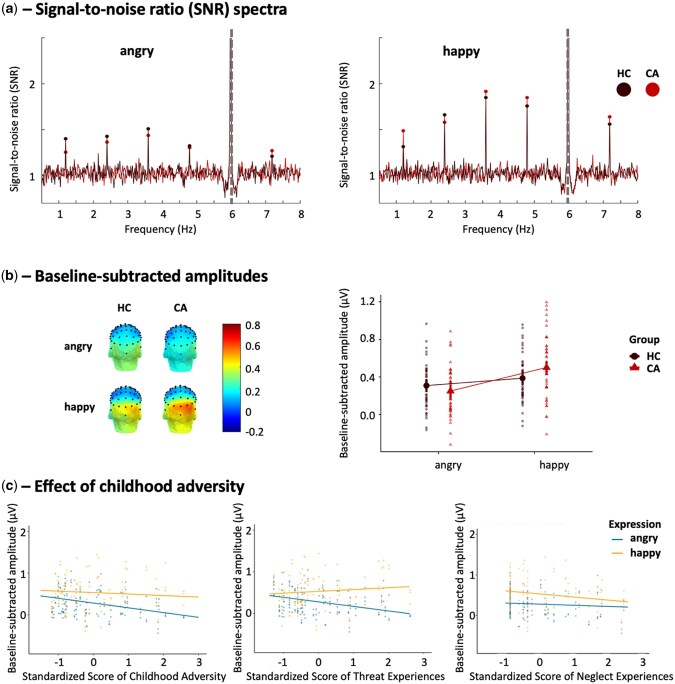
Discriminative responses to angry and happy faces from neutral faces in the oddball paradigm. (a) SNR EEG spectra of the oddball experiment averaged across the three ROIs for each facial expression and each participant group. HC responses are depicted in black, CA responses in red. The significant first five harmonics are displayed. The dashed line indicates the 6 Hz base rate response. (b) Left: Scalp distributions of the EEG signal based on the baseline-subtracted amplitudes in µV for each expression and each group. Right: Baseline-subtracted amplitudes summed across the five harmonics for each expression and each group. (c) Left: The impact of individual differences in childhood adversity on the discriminative responses of angry-neutral (β = −0.11, SE = 0.05, 95% CI, −0.21 to −0.02) and happy-neutral faces (β = −0.04, SE = 0.05, 95% CI, −0.13 to 0.06) within the adversity group. Right: The impact of individual differences in threat experiences on the discriminative responses of angry-neutral (β = −0.11, SE = 0.05, 95% CI, −0.21 to −0.01) and happy-neutral (β  =  0.04, SE = 0.05, 95% CI, −0.06 to 0.15) faces within the adversity group. Standardized scores were used.

Visualization of the SNR spectra showed clear peaks at the oddball frequency and harmonics for both groups and both emotional expressions, indicating automatic neural discrimination between the emotional and neutral faces (Fig. 2a). Model_1,_ contrasting both participant groups, revealed a main effect of expression (β = −0.08, SE = 0.03, *P* = .015, 95% CI, −0.14 to −0.02), a main effect of group (β = 0.11, SE = 0.05, *P* = .024, 95% CI, 0.02–0.21), and a significant group by expression interaction effect (β = −0.17, SE = 0.05, *P* < .001, 95% CI, −0.26 to 0.08). Post hoc analysis of the interaction effect showed that angry faces elicited a lower discriminative response than happy faces, but only in the CA group (mean_angry_ = 0.25, s.e. = 0.04 vs mean_happy_ = 0.50, s.e. = 0.04; β = −0.25, SE = 0.03, 95% CI, −0.33 to −0.17) and not in the HC group (mean_angry_ = 0.31, s.e. = 0.04 vs mean_happy_ = 0.39, s.e. = 0.04; β = −0.08, SE = 0.03, 95% CI, −0.16 to 0.01) (Fig. 2b), while the two groups did not differ in angry-neutral face discrimination (β  =  0.06, SE = 0.05, 95% CI, −0.07 to 0.19) and happy-neutral discrimination (β = −0.11, SE = 0.05, 95% CI, −0.24 to 0.02). In other words, participants who experienced adversity were less sensitive to discriminating angry versus neutral faces than discriminating happy versus neutral faces.

Model_2_ investigated this pattern within the CA group using a continuous adversity measure while controlling for individual differences in psychopathology, and again showed the main effect of expression (β = −0.25, SE = 0.03, *P* < .001, 95% CI, −0.32 to −0.19), and the adversity by expression interaction effect (β = −0.08, SE = 0.04, *P* = .044, 95% CI, −0.16 to −0.00). This indicates that a higher level of adversity was uniquely related to decreased angry-neutral discrimination relative to happy-neutral discrimination, which was mainly driven by the association of adversity with decreased angry-neutral discrimination (β = −0.11, SE = 0.05, 95% CI, −0.21 to −0.02) but not with happy-neutral discrimination (β = −0.04, SE = 0.05, 95% CI, −0.13 to 0.06) (Fig. 2c, left). Individual variability in severity of symptomatology did not modulate the neural sensitivity for these facial expressions.

Model_3_, disentangling the impact of both adversity dimensions, demonstrated that the reduced angry-neutral relative to happy-neutral face discrimination in participants with adversity was exclusively driven by individual differences in threat (β = −0.15, SE = 0.04, *P* = .001, 95% CI, −0.24 to −0.07), but not neglect experiences (β  =  0.05, SE = 0.04, *P* = .205, 95% CI, −0.03 to 0.12). Again, post hoc tests showed that the response pattern was mainly driven by the association of threat experiences with decreased angry-neutral face discrimination (β = −0.11, SE = 0.05, 95% CI, −0.21 to −0.01) but not with happy-neutral face discrimination (β = 0.04, SE = 0.05, 95% CI, −0.06 to 0.15) (Fig. 2c, right). Full statistical results of the three models are reported in Table S6.

### Neural responses and visual looking patterns towards angry vs. Neutral faces in the multi-input paradigm

#### Neural responses

**Figure 3.**
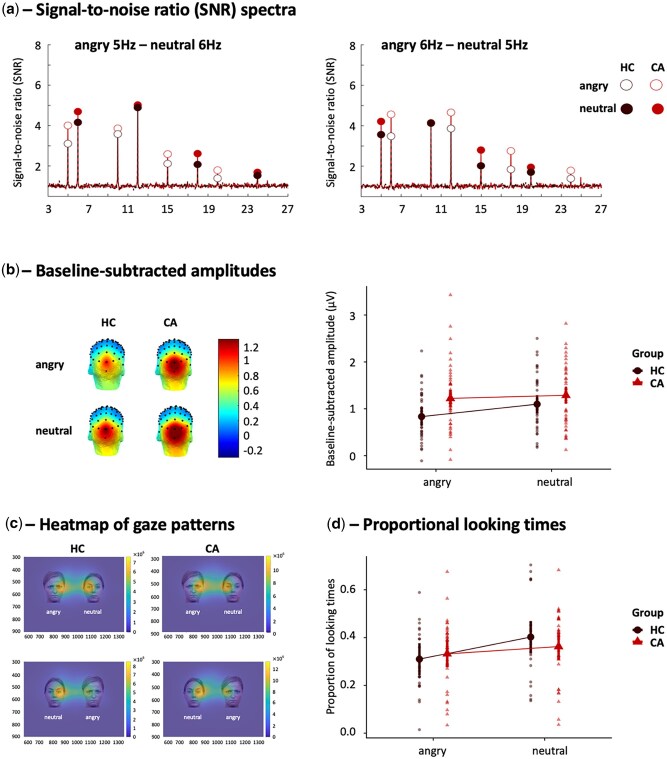
Neural responses and visual looking towards simultaneously presented angry and neutral faces in the multi-input paradigms. (a) SNR spectra of the neural responses at the MO region for each expression and each group at the 5 Hz and 6 Hz presentation rate. The significant first four harmonics are displayed. HC responses are depicted in black, CA responses in red. Responses for angry faces are depicted with open circles, responses for neutral faces with filled circles. (b) Left: Scalp distributions of the EEG signal based on the baseline-subtracted amplitudes in µV for each expression and each group. Right: Baseline-subtracted amplitudes at the MO region summed across the four harmonics for each expression and each group. (c) Heatmaps of gaze patterns averaged over participants of each group. Angry faces are presented in the left visual field on the top row and in the right visual field on the bottom one. The orange colouring indicates areas with longer total looking times. The x- and y-axis scales represent the position of the plotting area in relation to the eye-tracking recording screen (1920 × 1080 pixels). (d) The average proportion of looking times to angry and neutral faces for each of the groups.

Visualization of the SNR spectra showed clear peaks at each stimulation frequency and harmonics (Fig. 3a), centred around the medial occipital cortex (Fig. 3b, left). Statistical analysis of Model_1_ (contrasting both groups) revealed a main effect of expression (β = −0.26, SE = 0.06, *P* < .001, 95% CI, −0.39 to −0.14), and a significant group by expression interaction effect (β  =  0.20, SE = 0.09, *P* = .026, 95% CI, 0.02–0.37), indicating that controls showed higher neural responses to neutral relative to angry faces (mean_neutral_ = 1.10, s.e. = 0.10 vs mean_angry_ = 0.83, s.e. = 0.10; β  =  0.27, SE = 0.06, 95% CI, 0.10–0.43), whereas participants with adversity showed no difference in neural response to the two categories of faces (mean_neutral_ = 1.29, s.e. = 0.09 vs mean_angry_ = 1.22, s.e. = 0.09; β  =  0.07, SE = 0.06, 95% CI, −0.09 to 0.22; Fig. 3b, right). In addition, larger responses to angry faces were detected in the CA than in the HC group (β  =  0.39, SE = 0.13, 95% CI, 0.04–0.74), whereas no group difference was observed for neutral faces (β  =  0.19, SE = 0.13, 95% CI, −0.16 to 0.54). Investigating these associations at a more fine-grained continuous level in the CA group, however, did not reveal any significant associations among individual differences in general adversity exposure (Model_2_) or threat experiences (Model_3_) on the one hand, and the neural responses on the other. Full statistical results of the three models are reported in Table S9.

#### Visual looking patterns

Figure 3c displays heatmaps of gaze patterns, revealing a preferential looking towards neutral faces, as supported by a main effect of expression (β = −0.09, SE = 0.05, *P* = .045, 95% CI, −0.18 to −0.00), i.e. longer looking times to neutral than to angry faces. Contrary to our hypothesis, no group or group by expression effect was observed, indicating a general preferential looking towards neutral faces. Similar to the neural data, a more fine-grained analysis of adversity at a continuous level did not yield any significant associations. Full statistical results are reported in Table S11. As expected (Vettori et al. 2020), individual differences in preferential looking times and neural responses were highly correlated across both groups, *r*_(62)_ = 0.73, *P* < .001.

## Discussion

Using two visual frequency-tagging EEG paradigms in combination with eye-tracking, the current study objectively quantified the neural discrimination of angry and happy faces from neutral faces, as well as the neural responses to and preferential looking patterns towards simultaneously presented angry versus neutral faces in youth with and without childhood adversity experiences and concurrent psychiatric symptoms. Contrary to our hypothesis, results revealed reduced discrimination of angry faces from neutral faces in youth with adversity, and this pattern was related to individual differences in the extent of threat experiences, but not neglect experiences or psychiatric symptoms. When neutral and angry faces were presented simultaneously in competition with each other, we observed indistinct neural responses to angry and neutral faces in youth with adversity, while controls showed higher neural responses to neutral faces. Higher neural responses to angry faces were also observed in youth with childhood adversity relative to controls. Together, these findings suggest increased neural sensitivity for threatening information in adversity, as well as reduced neural threat-safety discrimination particularly in relation to threat experiences.

### Lower angry-neutral discrimination is observed in the adversity group and this pattern uniquely relates to youth’s threat but not neglect experiences

We observed lower angry-neutral discrimination in the adversity group. This finding contrasts with our initial hypothesis (i.e. increased angry-neutral discrimination in youth with adversity). Yet, the threat-related information processing bias in victims exposed to adversity may not only imply a hypervigilance towards objective threat information but can also involve a tendency to perceive neutral/safe and ambiguous information as being threatening. For instance, adolescents exposed to bullying have been found to mistake neutral faces more often as angry compared to their unexposed peers (Franzen et al. 2021). The reduced angry-neutral face discrimination in this study may thus indicate an already negative perception of neutral faces in youth exposed to childhood adversity, thus less distinct from angry faces. The results of the multi-input experiment further support this interpretation: when angry and neutral faces were presented simultaneously, controls showed higher neural responses to neutral than to angry faces, whereas youth with childhood adversity exhibited similar neural responses to both facial expressions. Consistent with this pattern, in a partially overlapping sample as reported here, we observed reduced discrimination between threat and safe cues during fear learning in youth with adversity compared to controls, primarily driven by heightened risk ratings of safe cues (Qiao et al. 2025).

We further found that individual differences in adversity exposure, more specifically threat but not neglect experiences, were related to the reduced angry-neutral face discrimination. This supports the hypothesis that the two dimensions of adversity exert a distinct impact: while deprivation, including neglect, may mainly impact cognitive development, such as language and executive functioning, threat may specifically impact emotional processing, such as threat detection and aversive learning (McLaughlin et al. 2014, 2019). Interestingly, in a parallel report on this same youth sample, threat but not neglect experiences were also found to be associated with reduced threat-safety discrimination during more complex scene processing, i.e. reduced neural discrimination of negative versus neutral social scenes (Qiao et al. 2025). Furthermore, a similar pattern of reduced angry-neutral face discrimination in relation to adversity exposure has also been observed in our parallel study in a general adolescent sample (12–16 years) (Samaey et al. 2024), even though, here, no specific effect of threat versus neglect was detected, potentially due to less neglect exposure. Thus, throughout two different populations and throughout multiple visual stimuli, our results consistently highlight the impact of childhood adversity, particularly threat experiences, on reduced threat-safety discrimination.

### Participants with adversity process simultaneously presented neutral and angry faces to a similar extent and show higher neural salience towards angry faces than controls

By presenting streams of angry versus neutral faces simultaneously, we found that controls showed higher neural responses to neutral as compared to angry faces, whereas youth with adversity showed similar neural responses to both face categories. This supports the findings of the oddball experiment that individuals with adversity perceive less contrast between angry versus neutral faces.

We further found that youth with adversity showed significantly higher EEG responses to angry faces than controls, supporting a neural salience bias towards angry faces in relation to childhood adversity. However, even though both modalities were highly correlated, this attentional bias at the neural level was not observable in the visual looking patterns monitored by eye-tracking. Supplementary analyses demonstrated that this discrepancy could not be attributed to any differences in size or composition of the participant groups, showing that the attentional bias at the neural level remained while only including subjects who also had eye-tracking data. This suggests that the frequency-tagging EEG approach is more sensitive in capturing more subtle adversity-related differences in facial expression processing, possibly because it captures both overt and covert attentional processing and because responses are unambiguously tagged to the presentation frequency of the stimuli (Vettori et al. 2020).

### No association of facial emotion processing with the severity of symptomatology

Although we found that childhood adversity, particularly threat exposure, was associated with both the presence of psychiatric symptoms and altered neural discrimination of facial expressions, no direct association between neural responses to facial expressions and the presence of symptomatology was observed. This is inconsistent with a previous finding in children (7–12 years): in a relatively small sample (16 physically abused and 14 non-abused children), Shackman and colleagues (Shackman et al. 2007) found that the association between parent-reported abusive behaviours and children’s reports of anxiety symptoms was mediated by ­children’s neural responses towards their mothers’ angry faces. However, considering the differences in sample characteristics (potential developmental effect) and methodological approach, it is difficult to compare this previous observation with our current findings. Our findings are consistent with previous studies suggesting an effect of childhood adversity above and beyond current psychiatric symptoms. For instance, the observed atypicalities in facial emotion processing in adults at high-risk for psychosis have been demonstrated to be related to their early adverse experiences (Tognin et al. 2020), and altered neural responses to emotional faces versus shapes were uniquely observed in adult victims of adversity who present internalizing psychopathology relative to both non-victims with internalizing psychopathology and healthy controls (Peters et al. 2019).

Importantly, from a clinical perspective, difficulties in discriminating threatening from non-threatening information in victims of childhood adversity may make them misinterpret ambiguous or even safe situations in daily life, thereby eventually putting them at risk for future adverse social experiences. On the other hand, they may miss out on the opportunity to establish and develop social relationships in the first place, resulting in social stress and thinning and ultimately contributing to psychiatric risk [see Mccrory et al. (2022) for a neurocognitive social transactional model of childhood adversity].

### Limitations

Our study has some limitations. First, our measures of childhood adversity rely on retrospective self-report by youth, which entails a possibility of recall bias. Retrospective reports of adverse experiences have been shown to agree only moderately with prospective reports, and to be rather associated with subjectively reported poor life outcomes than with objectively assessed outcomes (Reuben et al. 2016). Moreover, we only included emotional and physical neglect in the deprivation dimension, whereas other categories (such as institutionalization) may possibly also affect facial emotion processing. Timing of exposure has also been suggested to influence the impacts of adversity (Saarinen et al. 2021), which is not specified in our study. Second, although we aimed for participants with emerging psychiatric symptoms in our adversity group, it cannot be excluded that some of them might already have met full diagnostic criteria or be in treatment. While dimensional analyses demonstrated a unique effect of threat exposure on reduced angry-neutral face discrimination independent of symptomatology, it would have been more convincing to demonstrate this in a group of adversity-exposed individuals without any psychiatric symptoms to highlight the unique effects of adversity versus psychopathology. Third, our samples were skewed in terms of sex, with less male participants, which is however representative of adversity populations, as women have greater exposure to adverse events than men (Giano et al. 2020). Although there is evidence showing sex differences in facial expression processing, no significant effect of sex as a covariate was observed in our study. Fourth, our design only concerns cross-sectional data. While no association was observed between altered facial emotion processing and current symptomatology, it is unknown whether atypicalities in facial emotion processing could predict the transition to a psychiatric disorder in the future.

## Conclusion

Taking advantage of the objective and implicit nature of frequency-tagging EEG, the current study revealed that childhood adversity, and more specifically threat experiences, relates to deficits in automatically discriminating angry from neutral faces and the enhanced neural responses towards angry faces. The observed alterations in emotion processing were not associated with the present symptoms. Longitudinal designs are informative about whether altered facial expression processing following childhood adversity could predict the trajectories of symptom development.

## Supplementary Material

nsaf105_Supplementary_Data

## Data Availability

The data used in this study are available on formal request from the corresponding author.

## References

[nsaf105-B1] Aas M , Kauppi K, Brandt CL et al Childhood trauma is associated with increased brain responses to emotionally negative as compared with positive faces in patients with psychotic disorders. Psychol Med 2017;47:669–79. 27834153 10.1017/S0033291716002762

[nsaf105-B2] Addolorato G , Ancona C, Capristo E et al State and trait anxiety in women affected by allergic and vasomotor rhinitis. J Psychosom Res 1999;46:283–9. 10193919 10.1016/s0022-3999(98)00109-3

[nsaf105-B3] Annie B , Turgeon J, Blais C et al Emotion recognition in adults with a history of childhood maltreatment: a systematic review. Trauma Violence Abuse 2023;24:278–94. 34238064 10.1177/15248380211029403 PMC9660286

[nsaf105-B4] Baldwin JR , Wang B, Karwatowska L et al Childhood maltreatment and mental health problems: a systematic review and meta-analysis of quasi-experimental studies. Am J Psychiatry 2023;180:117–26. 10.1176/appi.ajp.20220174 PMC7614155 36628513

[nsaf105-B5] Baudouin JY , Poncet F, Polinori A et al Task-related modulation of facial expression processing: an FPVS-EEG study. Emotion 2023;23:2399–419. 10.1037/emo0001223 36996175

[nsaf105-B6] Beck AT , Steer RA, Brown GK. Manual for the Beck Depression Inventory-II. San Antonio, TX: Psychological Corporation, 1996.

[nsaf105-B7] Bernstein DP , Fink L. Childhood Trauma Questionnaire: A Retrospective Self-Report Manual. San Antonio: TX: The Psychological Corporation, 1998.

[nsaf105-B8] Bertó C , Ferrin M, Barberá M et al Abnormal emotional processing in maltreated children diagnosed of complex posttraumatic stress disorder. Child Abuse Negl 2017;73:42–50. 28945995 10.1016/j.chiabu.2017.09.020

[nsaf105-B9] Bodenschatz CM , Skopinceva M, Ruß T et al Attentional bias and childhood maltreatment in clinical depression—an eye-tracking study. J Psychiatr Res 2019;112:83–8. 10.1016/j.jpsychires.2019.02.025 30870713

[nsaf105-B10] Briggs-Gowan MJ , Pollak SD, Grasso D et al Attention bias and anxiety in young children exposed to family violence. J Child Psychol Psychiatry 2015;56:1194–201. 26716142 10.1111/jcpp.12397 PMC4697277

[nsaf105-B11] Catalan A , Artaza MGD, Bustamante S et al Differences in facial emotion recognition between first episode psychosis, borderline personality disorder and healthy controls. PLoS One 2016;11:e0160056. 10.1371/journal.pone.0160056 PMC4965014 27467692

[nsaf105-B12] Coll MP , Murphy J, Catmur C et al The importance of stimulus variability when studying face processing using fast periodic visual stimulation: a novel ‘mixed-emotions’ paradigm. Cortex 2019;117:182–95. 10.1016/j.cortex.2019.03.006 30986633

[nsaf105-B13] Cotter J , Granger K, Backx R et al Social cognitive dysfunction as a clinical marker: a systematic review of meta-analyses across 30 clinical conditions. Neurosci Biobehav Rev 2018;84:92–9. 29175518 10.1016/j.neubiorev.2017.11.014

[nsaf105-B14] da Silva Ferreira GC , Crippa JAS, de Lima Osório F. Facial emotion processing and recognition among maltreated children: a systematic literature review. Front Psychol 2014;5:1460. 10.3389/fpsyg.2014.01460 PMC4269127 25566138

[nsaf105-B15] Dennis CL , Coghlan M, Vigod S. Can we identify mothers at-risk for postpartum anxiety in the immediate postpartum period using the State-Trait anxiety inventory? J Affect Disord 2013;150:1217–20. 10.1016/j.jad.2013.05.049 23764383

[nsaf105-B16] Doretto V , Scivoletto S. Effects of early neglect experience on recognition and processing of facial expressions: a systematic review. Brain Sci 2018;8:10. 10.3390/brainsci8010010 PMC5789341 29316648

[nsaf105-B17] Dzhelyova M , Jacques C, Dormal G et al High test-retest reliability of a neural index of rapid automatic discrimination of unfamiliar individual faces. Vis Cogn 2019;27:127–41.

[nsaf105-B18] Dzhelyova M , Jacques C, Rossion B. At a single glance: fast periodic visual stimulation uncovers the spatio-temporal dynamics of brief facial expression changes in the human brain. Cereb Cortex 2017;27:4106–23. 27578496 10.1093/cercor/bhw223

[nsaf105-B19] Finkelhor D , Ormrod RK, Turner HA et al Measuring poly-victimization using the juvenile victimization questionnaire. Child Abuse Negl 2005;29:1297–312. 10.1016/j.chiabu.2005.06.005 16274741

[nsaf105-B20] Franzen M , de Jong PJ, Veling W et al Victims of bullying: emotion recognition and understanding. Front Psychol 2021;12:729835. 10.3389/fpsyg.2021.729835 PMC8551375 34721191

[nsaf105-B21] Giano Z , Wheeler DL, Hubach RD. The frequencies and disparities of adverse childhood experiences in the U.S. BMC Public Health 2020;20:1327. 10.1186/s12889-020-09411-z PMC7488299 32907569

[nsaf105-B22] Harms MB , Martin A, Wallace GL. Facial emotion recognition in autism spectrum disorders: a review of behavioral and neuroimaging studies. Neuropsychol Rev 2010;20:290–322. 10.1007/s11065-010-9138-6 20809200

[nsaf105-B23] Humphreys KL , Kircanski K, Colich NL et al Attentional avoidance of fearful facial expressions following early life stress is associated with impaired social functioning. J Child Psychol Psychiatry 2016;57:1174–82. 27457566 10.1111/jcpp.12607 PMC5030156

[nsaf105-B24] James G , Witten D, Hastie T et al An Introduction to Statistical Learning with Application in R. 2nd ed. New York, NY: Springer, 2021.

[nsaf105-B25] Kirkham EJ , Levita L. Early life stress is associated with reduced avoidance of threatening facial expressions. Dev Psychopathol 2020;32:1059–67. 10.1017/S095457941900110X 31405395

[nsaf105-B26] Langner O , Dotsch R, Bijlstra G et al Presentation and validation of the radboud faces database presentation and validation of the radboud faces database. Cogn Emot 2010;24:1377–88.

[nsaf105-B27] Lenth R , Singmann H, Love J et al Estimated marginal means, aka least-squares means. R package version 1.7.3, 2022.

[nsaf105-B28] Liu-Shuang J , Norcia AM, Rossion B. An objective index of individual face discrimination in the right occipito-temporal cortex by means of fast periodic oddball stimulation. Neuropsychologia 2014;52:57–72. 24200921 10.1016/j.neuropsychologia.2013.10.022

[nsaf105-B29] Mccrory E , Foulkes L, Viding E. Social thinning and stress generation after childhood maltreatment : a neurocognitive social transactional model of psychiatric vulnerability. Lancet Psychiatry 2022;9:828–37. 35926524 10.1016/S2215-0366(22)00202-4

[nsaf105-B30] Mclaughlin KA , Lambert HK. Child trauma exposure and psychopathology: mechanisms of risk and resilience. Curr Opin Psychol 2017;14:29–34. 10.1016/j.copsyc.2016.10.004 PMC5111863 27868085

[nsaf105-B31] McLaughlin KA , Sheridan MA. Beyond cumulative risk: a dimensional approach to childhood adversity. Curr Dir Psychol Sci 2016;25:239–45. 27773969 10.1177/0963721416655883 PMC5070918

[nsaf105-B32] McLaughlin KA , Sheridan MA, Lambert HK. Childhood adversity and neural development: deprivation and threat as distinct dimensions of early experience. Neurosci Biobehav Rev 2014;47:578–91. 25454359 10.1016/j.neubiorev.2014.10.012 PMC4308474

[nsaf105-B33] McLaughlin KA , Weissman D, Bitrán D. Childhood adversity and neural development: a systematic review. Annu Rev Dev Psychol 2019;1:277–312. 32455344 10.1146/annurev-devpsych-121318-084950 PMC7243625

[nsaf105-B34] Medeiros M , Tatiana A, Kha C et al Facial emotion recognition in maltreated children: a systematic review. J Child Fam Stud 2020;29:1493–509.

[nsaf105-B35] Naumann S , Bayer M, Dziobek I. Enhanced neural sensitivity to brief changes of happy over angry facial expressions in preschoolers: a fast periodic visual stimulation study. Psychophysiology 2025;62:e14725. 10.1111/psyp.14725 PMC11775875 39558668

[nsaf105-B36] Nelson CA , Westerlund A, McDermott JM et al Emotion recognition following early psychosocial deprivation. Dev Psychopathol 2013;25:517–25. 10.1017/S0954579412001216 PMC4127426 23627960

[nsaf105-B37] Pena-garijo J , Lacruz M, Masanet MJ et al Specific facial emotion recognition deficits across the course of psychosis: a comparison of individuals with low-risk, high-risk, first-episode psychosis and multi-episode schizophrenia-spectrum disorders. Psychiatry Res 2023;320:115029. 10.1016/j.psychres.2022.115029 36586376

[nsaf105-B38] Peters AT , Burkhouse KL, Kinney KL et al The roles of early-life adversity and rumination in neural response to emotional faces amongst anxious and depressed adults. Psychol Med 2019;49:2267–78. 10.1017/S0033291718003203 PMC6513724 30419983

[nsaf105-B39] Petersen SE , Posner MI. The attention system of the human brain: 20 years after. Annu Rev Neurosci 2012;35:73–89. 22524787 10.1146/annurev-neuro-062111-150525 PMC3413263

[nsaf105-B40] Pinheiro J , Bates D, Team RC. nlme: Linear and Nonlinear Mixed Effects Models. R package version 3.1-166, 2024.

[nsaf105-B41] Pollak SD , Cicchetti D, Hornung K et al Recognizing emotion in faces: developmental effects of child abuse and neglect. Dev Psychol 2000;36:679–88. 10.1037/0012-1649.36.5.679 10976606

[nsaf105-B42] Pollak SD , Sinha P. Effects of early experience on children’s recognition of facial displays of emotion. Dev Psychol 2002;38:784–91. 12220055 10.1037//0012-1649.38.5.784

[nsaf105-B43] Pollak SD , Tolley-Schell SA. Selective attention to facial emotion in physically abused children. J Abnorm Psychol 2003;112:323–38. 10.1037/0021-843x.112.3.323 12943012

[nsaf105-B44] Poncet F , Baudouin JY, Dzhelyova MP et al Rapid and automatic discrimination between facial expressions in the human brain. ­Neuropsychologia 2019;129:47–55. 10.1016/j.neuropsychologia.2019.03.006 30885642

[nsaf105-B45] Posner MI , Petersen SE. The attention system of the human brain. Annu Rev Neurosci 1990;13:25–42. 10.1146/annurev.ne.13.030190.000325 2183676

[nsaf105-B46] Qiao Z , Van der Donck S, Mazereel V et al Implicit neural sensitivity for negatively valued social and non-social visual scenes in young adults exposed to childhood adversity. Psychol Med 2025;55:e37. 39930797 10.1017/S0033291725000029 PMC12017353

[nsaf105-B47] Qiao Z , Donck S, Van Der Moerkerke M et al Frequency-Tagging EEG of superimposed social and non-social visual stimulation streams provides no support for social salience enhancement after intranasal oxytocin administration. Brain Sci 2022;12:1224. 36138960 10.3390/brainsci12091224 PMC9496939

[nsaf105-B48] Qiao Z , Samaey C, Jennen L et al Reduced threat-safety discrimination and generally enhanced generalization responses in adversity exposed youth with emerging psychiatric symptoms. BMC Psychiatry 2025;25:797. 40830778 10.1186/s12888-025-07254-9 PMC12362923

[nsaf105-B49] R Core Team. R: A Language and Environmentfor Statistical Computing. R Foundation for Statistical Computing; Vienna, Austria, 2022.

[nsaf105-B50] Reuben A , Moffitt TE, Caspi A et al Lest we forget: comparing retrospective and prospective assessments of adverse childhood experiences in the prediction of adult health. J Child Psychol Psychiatry 2016;57:1103–12. 27647050 10.1111/jcpp.12621 PMC5234278

[nsaf105-B51] Rutherford MD , McIntosh DN. Rules versus prototype matching: Strategies of perception of emotional facial expressions in the autism spectrum. J Autism Dev Disord 2007;37:187–96. 16897386 10.1007/s10803-006-0151-9

[nsaf105-B52] Saarinen A , Keltikangas-järvinen L, Jääskeläinen E et al Early adversity and emotion processing from faces: a meta-analysis on behavioral and neurophysiological responses. Biol Psychiatry Cogn Neurosci Neuroimaging 2021;6:692–705. 33486133 10.1016/j.bpsc.2021.01.002

[nsaf105-B53] Samaey C , Van der Donck S, Lecei A et al Between Faces: childhood adversity is associated with reduced threat-safety discrimination during facial expression processing in adolescence. BMC Medicine 2024;22:382. 10.1186/s12916-024-03610-w PMC11389258 39256825

[nsaf105-B54] Samaey C , Van der Donck S, van Winkel R et al Facial expression processing across the autism–psychosis spectra: a review of neural findings and associations with adverse childhood events. Front Psychiatry 2020;11:592937–17. 10.3389/fpsyt.2020.592937 PMC7691238 33281648

[nsaf105-B55] Savill M , D’Ambrosio J, Cannon TD et al Psychosis risk screening in different populations using the prodromal questionnaire: a systematic review. Early Interv Psychiatry 2018;12:3–14. 10.1111/eip.12446 PMC5812357 28782283

[nsaf105-B56] Schäfer JL , Rohde LA, Mclaughlin KA et al Threat and deprivation are associated with distinct aspects of cognition, emotional processing, and psychopathology in children and adolescents. Dev Sci 2023;26:e13267. 35417607 10.1111/desc.13267 PMC10028496

[nsaf105-B57] Shackman JE , Shackman AJ, Pollak SD. Physical abuse amplifies attention to threat and increases anxiety in children. Emotion 2007;7:838–52. 18039053 10.1037/1528-3542.7.4.838

[nsaf105-B59] Spielberger CD , Gorsuch RL, Lushene R et al State-Trait Anxiety Inventory for Adults. Mind Garden 1983.

[nsaf105-B60] Tognin S , Catalan A, Modinos G et al; EU-GEI High Risk Study. Emotion recognition and adverse childhood experiences in individuals at clinical high risk of psychosis. Schizophr Bull 2020;46:823–33. 32080743 10.1093/schbul/sbz128 PMC7345818

[nsaf105-B61] Van der Donck S , Dzhelyova M, Vettori S et al Fast periodic visual stimulation EEG reveals reduced neural sensitivity to fearful faces in children with autism. J Autism Dev Disord 2019;49:4658–73. 31468275 10.1007/s10803-019-04172-0 PMC6813754

[nsaf105-B62] Van der Donck S , Dzhelyova M, Vettori S et al Rapid neural categorization of angry and fearful faces is specifically impaired in boys with autism spectrum disorder. J Child Psychol Psychiatry 2020;61:1019–29. 10.1111/jcpp.13201 PMC7496330 32003011

[nsaf105-B63] Van der Donck S , Moerkerke M, Dlhosova T et al Monitoring the effect of oxytocin on the neural sensitivity to emotional faces via frequency-tagging EEG: a double-blind, cross-over study. Psychophysiology 2022;59:e14026. 10.1111/psyp.14026 35150446

[nsaf105-B64] Van Nierop M , Viechtbauer W, Gunther N et al; Genetic Risk and OUtcome of Psychosis investigators. Childhood trauma is associated with a specific admixture of affective, anxiety, and psychosis symptoms cutting across traditional diagnostic boundaries. Psychol Med 2015;45:1277–88. 10.1017/S0033291714002372 25273550

[nsaf105-B65] Vettori S , Dzhelyova M, Van der Donck S et al Combined frequency-tagging EEG and eye tracking reveal reduced social bias in boys with autism spectrum disorder. Cortex 2020;125:135–48. 10.1016/j.cortex.2019.12.013 31982699

[nsaf105-B66] Whisman MA , Richardson ED. Normative data on the beck depression inventory—second edition (BDI-II) in college students. J Clin Psychol 2015;71:898–907. 10.1002/jclp.22188 25950150

