# Supplementary material for: Early threat experiences relate to reduced neural face discrimination in youth with emerging psychiatric symptoms: a frequency-tagging electroencephalography study
Source: Soc Cogn Affect Neurosci. 2025 Oct 14;20(1):nsaf105. doi: 10.1093/scan/nsaf105 (PMC12596126; doi:10.1093/scan/nsaf105)
Supplement: nsaf105_Supplementary_Data [file nsaf105_supplementary_data.zip › Supporting Information.docx]

**Supporting Information**

Contents

[Appendix S1: Participant exclusion criteria 2](#_Toc207790789)

[Appendix S2: Measurement of childhood adversity and symptoms 3](#_Toc207790790)

[Appendix S3: Stimuli 5](#_Toc207790791)

[Appendix S4: EEG and eye-tracking data recording 6](#_Toc207790792)

[Appendix S5: EEG and eye-tracking data preprocessing 7](#_Toc207790793)

[Appendix S6: Supplementary statistical analysis 11](#_Toc207790794)

[Appendix S7: Participant enrolment and demographic information 13](#_Toc207790795)

[Appendix S8: Supplementary results of the oddball paradigm 16](#_Toc207790796)

[Appendix S9: Supplementary results of the multi-input paradigm 20](#_Toc207790797)

**[References](#_Toc207790798)** [25](#_Toc207790798)

# Appendix S1: Participant exclusion criteria

The exclusion criteria are: being actively in psychotropic treatment, medical or neurological disorders impeding participation (i.e. refractory diabetes, disabling heart conditions, refractory epilepsy or neuromuscular diseases), current use (or use in the month preceding the study) of psychoactive substances, alcohol consumption >14 units for both men and women according to the NICE guidelines on harmful use (NICE Guidelines, 2010), active suicidality, autism spectrum disorder and intellectual disability.

# Appendix S2: Measurement of childhood adversity and symptoms

**Childhood adversity.** All measures were conducted in REDCap (<https://www.project-redcap.org/>). For adversity, a modified screening version of the Juvenile Victimization Questionnaire 2nd revision (Adult Retrospective Form, JVQ-R2; (Finkelhor *et al.*, 2005)) and 5 questions of the Emotional Neglect subscale of the Childhood Trauma Questionnaire (CTQ; (Bernstein and Fink, 1998)) were used to measure childhood adversity before 18 years old. A total of 33 items covered 7 categories of childhood adversity: peer and sibling victimization/bullying, physical abuse, emotional abuse, sexual abuse, physical neglect, emotional neglect and domestic violence. For each item, participants were asked to indicate the exposure to and frequency of (1=once, 2=seldom, 3=sometimes, 4=often, 5=very often) that experience. They were included into the CA group if they answered yes to (at least) one of the items in the categories peer and sibling victimization/bullying, domestic violence and emotional neglect, with a frequency of (at least) ‘sometimes’. They were also included in the CA group if they answered yes to one of the items in the categories physical abuse, physical neglect, emotional abuse and sexual abuse (at any frequency), or to one item of the emotional abuse category (“At any time in your life, did you get scared or feel really bad because grown-ups in your life called you names, said mean things to you, or said they didn’t want you?”) and one of the two items of the sexual abuse category (“Did anyone ever force you to look at his or her intimate parts by forcing or surprising you or by suddenly showing you?”, “Did anybody ever hurt your feelings by saying or writing something sexual about you or your body?”), with a frequency of at least “sometimes”, in line with a prior study in an independent sample (Croft *et al.*, 2019). During the test session, participants completed an interview with one of our two researchers, where we measured the endorsement and frequency of childhood adversity again. Based on this, a continuous score of CA was created by averaging the mean frequency of the seven adversity categories to indicate the general adversity exposure. We further averaged the mean frequency of physical and emotional neglect and the mean frequency of the other five adversity categories to indicate individual differences in neglect and threat experiences, separately.

**Symptoms.** Depressive symptoms were measured using the Beck Depression Inventory (BDI-II), with a predefined cut-off score of ≥ 11 indicating the presence of clinically relevant depression symptoms (Beck *et al.*, 1996; Whisman and Richardson, 2015). The trait scale of the State Trait Anxiety Inventory was used to measure anxiety and a cut-off score of ≥ 40 was used to indicate the presence of clinically relevant anxiety (Spielberger *et al.*, 1983; Addolorato *et al.*, 1999; Dennis *et al.*, 2013). Psychotic symptoms were measured using the Prodromal Questionnaire-16 version (PQ-16), with a cut-off score of ≥ 5 on the symptom scale or ≥ 8 on the distress scale to indicate the presence of clinically relevant psychotic symptoms (Savill *et al.*, 2018). During the testing session, participants again completed the same questionnaires to re-assess their symptoms during the testing period.

# Appendix S3: Stimuli

**Stimuli.** The stimuli for both paradigms consisted of facial images of 13 men and 13 women with angry, happy and neutral expressions, selected from the Radboud Faces Database (Langner *et al.*, 2010). All stimuli were sized at 300 × 300 pixels, with their mean pixel luminance and contrast equalized using a built-in function in *Sinstim* (a stimulus presentation software). During the oddball task, stimuli were displayed at the center of a 24-inch LCD screen (resolution: 2560 × 1440 pixels). In the multi-input paradigm, the two face images were positioned 310 pixels apart, centered symmetrically at coordinates (-155, 0) for the left image and (155, 0) for the right image. A screen with a 60 Hz frequency was used to ensure that the refresh rate was an integer multiple of the stimuli presentation frequencies. Using a custom-built Java script, stimuli were presented on the screen through sinusoidal contrast modulation on a light grey background. Participants were seated in front of the screen at a 60 cm viewing distance in a dimly lit room and were instructed to maintain a constant distance during data recording. The eye-tracking data of the multi-input paradigm was recorded simultaneously via a computer screen with a resolution of 1920 × 1080 pixels.

# Appendix S4: EEG and eye-tracking data recording

**EEG data recording.** The continuous EEG signal was sampled at 512 Hz for both paradigms using a BioSemi Active-Two amplifier system with 64 Ag/AgCl electrodes. Two additional electrodes (common mode sense and driven right leg) were utilized as reference and ground electrodes. Horizontal eye movements were captured by two external electrodes positioned at the outer canthi of the eyes and vertical eye movements were recorded by two external electrodes placed above and below the right eye.

**Eye tracking data recording.** During the multi-input EEG paradigm, eye gaze data was recorded at 120 Hz using a Tobii X3-120 screen-based remote eye tracker and Tobii Pro software (Tobii Pro). Following the standard calibration procedure of the Tobii X3-120 to ensure eye movements were correctly tracked, an additional calibration validation procedure (Vettori, Dzhelyova, *et al.*, 2020) was administered, where participants had to fixate on the center of ten consecutive fixation crosses appearing on different locations across the screen. This procedure allows us to obtain a subject-specific quantitative measure of data quality, including an index of error angle (mean and variance) and the resulting accuracy, by calculating the angle between the vectors to the displayed fixation cross versus the actual gaze point. These values were used when attributing gaze points to particular areas of interest (AOIs).

# Appendix S5: EEG and eye-tracking data preprocessing

**Preprocessing of EEG data.** Data preprocessing was conducted using Letswave 6 (https://www.letswave.org/) and MATLAB 2021a (MathWorks). Specifically, the raw EEG signal was cropped into segments with 4 s before and 6 s after each sequence, resulting in 70-s segments for the oddball paradigm and 40-s segments for the multi-input paradigm. Thereafter, the EEG data were bandpass filtered (0.1 to 100 Hz) using a fourth-order Butterworth filter and down sampled to 256 Hz. Noisy channels were linearly interpolated using the three spatially nearest electrodes (not more than 5% of the electrodes, i.e. three electrodes, were interpolated). All data segments were re-referenced to a common average reference. The preprocessed segments of the oddball paradigm were further cropped to contain an integer number of 1.1973 Hz (the actual presentation frequency of 1.2 Hz) cycles, starting from the beginning of the sequence until 59.3008 s (15,181 time bins). The resulting segments were averaged in the time domain for each expression (i.e. angry and happy sequences) for each participant and transformed into the frequency domain with a fast Fourier transformation (FFT), yielding a spectrum between 0 and 127.9747 Hz. For the multi-input paradigm, the preprocessed segments of were cropped to contain an integer number of 0.9978 Hz (the actual presentation frequency of 1 Hz) cycles (i.e. the largest common divisor of both 5 and 6 Hz), starting from the beginning of the sequence until 29.0625 s (7,440 time bins). For each participant, the resulting segments were averaged in the time domain per presentation rate for each stimulus type (angry 5Hz vs neutral 6Hz and angry 6Hz vs neutral 5Hz sequences) and transformed into the frequency domain, yielding a spectrum between 0 and 127.9656 Hz.


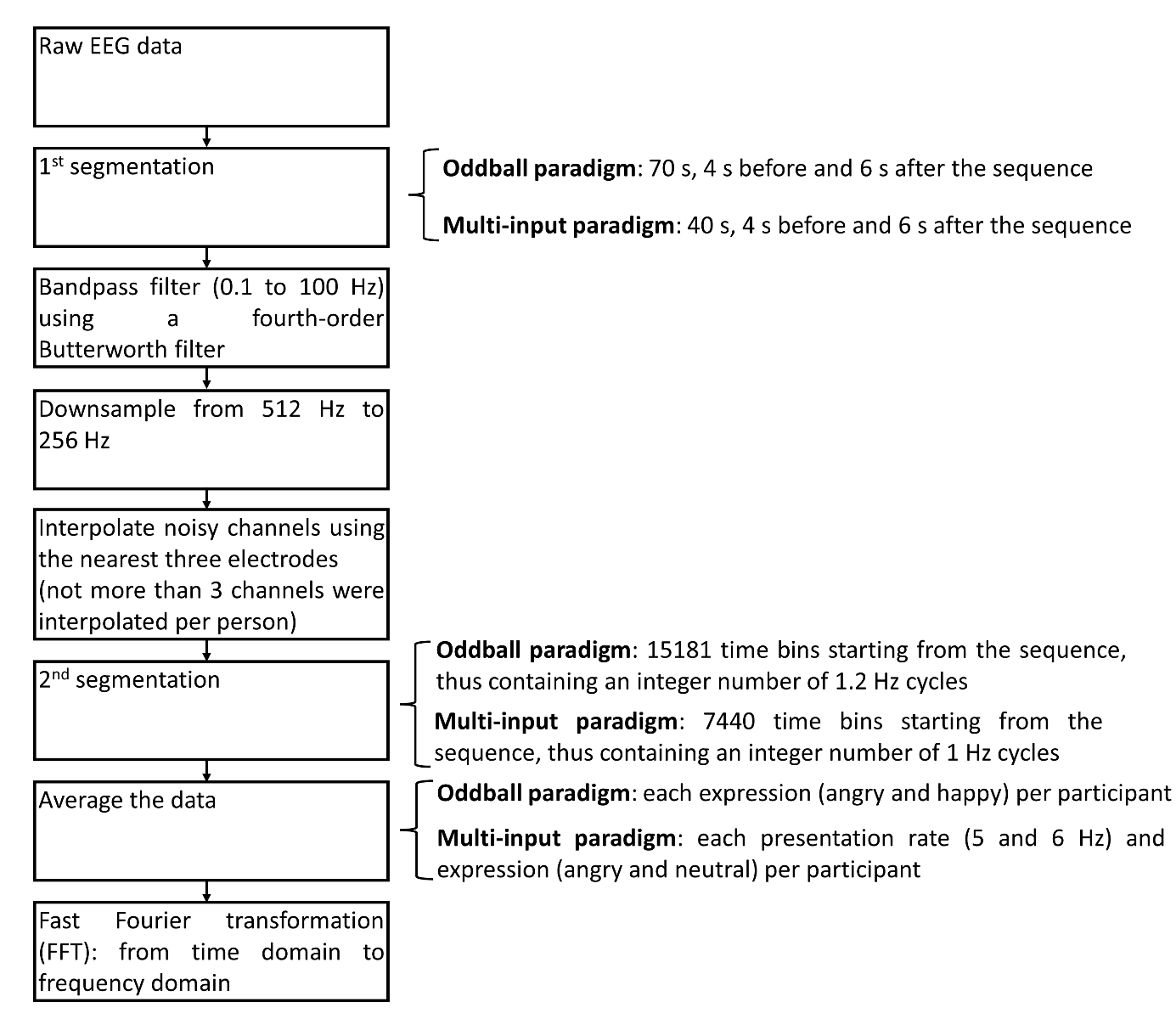


**Figure S1.** The flowchart of EEG data preprocessing.

**Eye tracking data.** In line with the previous study (Vettori, Van der Donck, *et al.*, 2020), eye-tracking data were analyzed using a series of custom-built scripts in Matlab (The Mathworks; https://github.com/TimVanWesemael/Fuzzy-AOI-EyeTracking). The I2MC algorithm (Hessels *et al.*, 2017) was utilized to filter the raw data: deleting random noise, interpolating missing data and identifying fixations. Two areas of interest (AOI; left and right visual region) were defined as the rectangular areas (200 × 300 pixels) where the two images of faces (removing the grey areas at the left and right side of the original images) were presented. In addition, to label all the fixation points that were not attributed to either left or right face AOI, we defined the area ‘outside AOI’.

The allocation of fixations to the AOIs was accomplished using a probability weighting approach while taking the subject-specific data quality into account (Vettori, Dzhelyova, *et al.*, 2020). Specifically, for every gaze point, a proportional score (i.e. between zero and one) was attributed to each AOI, which indicated the probability that the corresponding AOI effectively contained the recorded gaze point. The assignment of these proportional scores was based on a two-dimensional bell curve around the gaze point, with a standard deviation determined by the root-mean-square registered during calibration validation (Vettori, Van der Donck, *et al.*, 2020). Better data quality during calibration validation resulted in more concentrated sample points around the gaze point; poorer data quality resulted in more dispersed sample points. This algorithm proves to be a very reliable method, since it takes every gaze point into account, as well as the subject-specific data quality (Vettori, Dzhelyova, *et al.*, 2020; Van der Donck *et al.*, 2021). For each AOI, the duration of all fixation points was averaged over the four sequences of each expression condition (e.g. angry faces presented at either the left or right visual region). Proportional looking times for the left and right face AOIs, as well as looking time outside the AOIs, were calculated.

**Orthogonal task performance.** Both accuracy and reaction times were calculated to evaluate the participants’ performance. A key press was considered correct if it occurred in the 100 to 2000 ms time window following an actual color change. For the oddball paradigm, the proportions and reaction times of the correct responses were averaged across the four sequences, resulting in an overall accuracy and reaction times per expression (i.e. angry and happy) per participant. For the multi-input paradigm, those were averaged across the four sequences, resulting in an overall accuracy and reaction time per presentation rate (i.e. angry 5Hz vs. neutral 6 Hz, angry 6 Hz vs. neutral 5 Hz) per participant.

# Appendix S6: Supplementary statistical analysis

Linear mixed models (LMM) were performed using *R*-package *nlme*, version 3.1-163 (Pinheiro *et al.*, 2023), in *RStudio*, version 4.1.3 (R Core Team., 2022). Tukey-corrected post-hoc t-tests were performed using *R*-package *emmeans*, version 1.7.3 (Lenth *et al.*, 2022). The median absolute deviation was used to detect outliers.

**Orthogonal task performance.** We fitted separate model for accuracy and reaction times, with Expression [angry and happy] and Group [HC and CA] as the fixed factors for the oddball paradigm and Frequency [angry 5Hz vs. neutral 6 Hz, and angry 6 Hz vs. neutral 5 Hz] and Group as the fixed factors for the multi-input paradigm. A random intercept by subject was included to account for the clustered nature of the repeated measurement. Age and sex were added into the models as covariates.

**Neural responses to the visual base stimulation in the oddball paradigm.** Similar analyses steps were performed as those in the main text. Specifically, with the neural responses to the visual base stimulation as the dependent variable, we constructed the following three linear mixed models (LMMs): Model_1_, *y ~ age + sex + ROI + Group * Expression + (1|subject)*, with Group [HC vs. CA] as the between-subjects effect and Expression [angry vs. happy] and ROI [LOT, MO, ROT] as the within-subjects effects; Model_2_, *y ~ age + sex + Childhood adversity * Expression + Depression * Expression + Anxiety * Expression + Psychosis * Expression + (1|subject)*, with adversity and symptoms as continuous measures; Model_3_, *y ~ age + sex + Threat * Expression + Neglect * Expression + Depression * Expression + Anxiety * Expression + Psychosis * Expression + (1|subject)*, with threat and neglect experiences and symptoms as continuous measures.

**Supplementary analyses of neural responses in the multi-input paradigm.** The same model_1_ as in the main analyses was fitted while only including the subjects who also had eye-tracking data (N = 67; 28 HC and 39 CA).

**Supplementary analyses of reliability for neural responses.** Following the reviewer’s suggestion, we re-preprocessed the data and assessed the reliability of neural responses. For the oddball paradigm, the four sequences were divided into two sequences containing female face images (i.e. with the same sequence marker) and two sequences containing male face images (i.e. with the same sequence marker) for each condition (angry and happy). The data from the two female sequences and the two male sequences were averaged separately for angry and happy conditions. We then applied fast Fourier transformation (FFT) to the averaged data, pooled channels into regions of interest (ROIs) and performed baseline-subtraction. Finally, Pearson correlation was computed between responses to the two female sequences and to the two male sequences for each condition (angry and happy).

For the multi-input paradigm, the eight sequences were split into four sequences where angry faces were presented at 6 Hz and neutral faces at 5 Hz, and 4 sequences where angry faces were presented at 5 Hz and neutral faces at 6 Hz. The data were re-preprocessed following the same steps as in the oddball paradigm. Pearson correlation was then calculated between responses to faces presented at 5 Hz (four sequences) and those presented at 6 Hz (four sequences) for both neutral and angry conditions.

**Exploratory analyses.** We conducted exploratory analyses to investigate the effect of adversity and symptoms (i.e. as continuous measure), while not controlling for each other, on the responses within the CA group. Specifically, for the neural discriminative responses in the oddball paradigm, we constructed the following five models: model_supp1_ *y ~ age + sex + Childhood adversity * Expression + (1|subject)*, model_supp2_ *y ~ age + sex + Threat * Expression + Neglect * Expression + (1|subject),* model_supp3_ *y ~ age + sex + Depression * Expression + (1|subject)*, model_supp4_ *y ~ age + sex + Anxiety * Expression + (1|subject)*, and model_supp5_ *y ~ age + sex + Psychosis * Expression + (1|subject)*, with Expression [angry vs. happy] as the within-subjects effect. For the neural sensitivity and visual looking patterns measured in the multi-input paradigm, five similar models were fitted, with Expression [angry vs. neutral] as the within-subjects effect. Continuous scores of childhood adversity, neglect and threat experiences and symptoms during testing were used and all scores were standardized to have a mean of 0 and a standard deviation of 1 before being added into the models.

# Appendix S7: Participant enrolment and demographic information

**Participant recruitment.** For the oddball paradigm, EEG data was recorded for 47 controls and 52 participants with CA, but six CA participants were excluded (one due to aberrant screen frequency, and three with missing symptom measures and two with CA scores below the cutoff during re-assessment in the test session), resulting in 46 CA participants. For the multi-input paradigm, EEG data was recorded for 43 controls and 52 CA participants; exclusions included two controls (one with aberrant screen frequency and one with incorrect triggers) and the same six CA participants as in the oddball paradigm, leaving 41 controls and 46 CA participants. Eye-tracking data for the multi-input paradigm was only available for 34 controls and 47 CA participants due to technical issues. After excluding six controls (four for poor calibration accuracy, one with corrupted data, and one with aberrant screen frequency) and eight CA participants (three for poor calibration accuracy, one with corrupted data, and two with missing symptom measures and two with CA scores below the cutoff during re-assessment in the test session), the final sample comprised 28 controls and 39 CA participants.

**Demographic information.** Data of measures were downloaded from REDCap, version “ENGAGE_DATA_2022-11-06_1208”. Demographic and clinical information of the overall sample is reported in Table S1. Based on the sample included in the oddball paradigm, we found a good test-retest reliability (i.e. between measures during screening and testing) for childhood adversity (CA group: *r*_(44)_ = .84, *p* < .001), threat experiences (CA group: *r*_(44)_ = .81, *p* < .001), neglect experiences (CA group: *r*_(44)_ = .77, *p* < .001), depression (CA group: *r*_(44)_ = .82, *p* < .001; HC group: *r*_(45)_ = .58, *p* < .001), anxiety (CA group: *r*_(44)_ = .71, *p* < .001; HC group: *r*_(45)_ =.68, *p* < .001) and psychotic symptoms (CA group: *r*_(44)_ = .83, *p* < .001; HC group: *r*_(45)_ = .83, *p* < .001). We further checked the association of childhood adversity (the general adversity exposure and the two dimensions) with the presence of subclinical symptoms based on the measurement during the test session (see Table S2). The number of participants scoring above and below the predefined cut-off score for each category of childhood adversity is reported in Table S3.

| **Table S1**. Demographic and clinical information of the full participant sample during the test session (HC = 47, CA = 52). | | | | |
| --- | --- | --- | --- | --- |
| **Characteristics** | | HC | CA | *p* value |
| Sex, female/male | | 30 /17 | 35 /17 | 716 |
| Age, years: mean (s.d.) | | 21.3 (1.92) | 19.9 (2.22) | .001 |
| Childhood adversity, average score:  mean (s.d.) | Adversity | 0.03 (0.06) | 0.53 (0.42) | < .001 |
|  | Threat | 0.04 (0.07) | 0.49 (0.38) | < .001 |
|  | Neglect | 0.03 (0.11) | 0.63 (0.77) | < .001 |
| Symptoms^*^,  score: mean (s.d. | Depression | 2.85 (2.92) | 19.1(10.4) | < .001 |
|  | Anxiety | 30.5 (5.27) | 53 (10.3) | < .001 |
|  | Psychosis | 1.11 (1.43) | 5.22 (3.32) | < .001 |
| Legend: Childhood adversity was calculated as a continuous score by averaging the mean frequency scores across seven categories (peer and sibling victimization/bullying, physical abuse, physical neglect, emotional abuse, emotional neglect, sexual abuse and domestic violence), assessed using a modified version of the Juvenile Victimization Questionnaire 2nd revision (Adult Retrospective Form; JVQ-R2) and the Emotional Neglect subscale of the Childhood Trauma Questionnaire (CTQ). We derived two composite measures: neglect exposure (mean of physical and emotional neglect scores) and threat exposure (mean of the remaining five categories). Psychiatric symptoms were assessed with the Beck Depression Inventory (BDI-II) for depression, the trait scale of the State Trait Anxiety Inventory for anxiety, and the symptom scale of the Prodromal Questionnaire-16 (PQ-16) for psychotic symptoms. Group comparisons were performed using independent samples t-tests for continuous variables and chi-square tests for sex distribution.  ^*^Three participants with missing data. | | | | |

| **Table S2** Correlation between childhood adversity and symptoms. | | | | | | |
| --- | --- | --- | --- | --- | --- | --- |
|  | **Depression** | | **Anxiety** | | **Psychosis** | |
|  | *r* | *p* value | *r* | *p* value | *r* | *p* value |
| Childhood adversity | .43 | .003 | .43 | .003 | .46 | .001 |
| Threat | .49 | < .001 | .49 | < .001 | .50 | < .001 |
| Neglect | .22 | .147 | .22 | .141 | .26 | .084 |

| **Table S3** Number and percentage of CA participants who scored larger than zero for threat, neglect, threat and neglect, and each childhood adversity category, stratified by paradigm in the final analyzed samples. | | |
| --- | --- | --- |
|  | **The oddball and multi-input paradigm**  **EEG (n = 46)** | **The multi-input paradigm**  **Eye tracking (n = 39)** |
| Threat | 44 (95.65%) | 37 (94.87%) |
| Neglect | 29 (63.04%) | 25 (64.10%) |
| Threat and neglect | 27 (58.70%) | 23 (58.97%) |
| Bullying/victimization | 42 (91.30%) | 35 (89.74%) |
| Emotional abuse | 15 (32.61%) | 13 (33.33%) |
| Physical abuse | 10 (21.74%) | 8 (20.51%) |
| Sexual abuse | 17 (36.96%) | 15 (38.46%) |
| Domestic violence | 18 (39.13%) | 14 (35.90%) |
| Emotional neglect | 28 (60.87%) | 24 (61.54%) |
| Physical neglect | 9 (19.57%) | 9 (23.08%) |

# Appendix S8: Supplementary results of the oddball paradigm

**Orthogonal task performance.** For reaction times, results showed equal performances for the two groups and the two expression conditions. Accuracies were generally very high, yet, results showed lower accuracy in the CA relative to the HC group (mean_CA_ 95.8%, s.d. = 0.65% vs mean_HC_ 98.0%, s.d. = 0.64%; Table S4).

| **Table S4** Linear mixed model to assess the orthogonal task performance of both groups in the oddball paradigm. | | | | |
| --- | --- | --- | --- | --- |
|  | **β** | **SE** | **95% CI** | ***p*** |
| **Speed** | | | | |
| Age | -0.00 | 0.00 | -0.01 – 0.00 | 0.543 |
| Sex | -0.01 | 0.01 | -0.03 – 0.01 | 0.441 |
| Group | 0.01 | 0.01 | -0.01 – 0.03 | 0.336 |
| Expression | 0.00 | 0.00 | -0.00 – 0.01 | 0.554 |
| Group * Expression | 0.00 | 0.00 | -0.01 – 0.01 | 0.699 |
| **Accuracy** | | | | |
| Age | -0.00 | 0.00 | -0.01 – 0.00 | 0.235 |
| Sex | 0.01 | 0.01 | -0.01 – 0.03 | 0.421 |
| Group | -0.02 | 0.01 | -0.04 – -0.00 | **0.049** |
| Expression | 0.01 | 0.01 | -0.00 – 0.02 | 0.054 |
| Group * Expression | -0.00 | 0.01 | -0.02 – 0.01 | 0.599 |

**Neural responses to the visual base stimulation.** Neural responses to the visual base stimulation (i.e. 6 Hz) were quantified as the summed responses of six harmonics, i.e. 6, 12, 18, 24, 30 and 36 Hz. In line with previous reports(Van der Donck *et al.*, 2019; Van der Donck *et al.*, 2022), a linear mixed model (*y ~ age + sex + Group * Expression + ROI + (1|subject)*) was constructed and revealed lower responses in the LOT and ROT regions relative to the MO region and larger responses in the ROT region relative to the LOT region. The absence of any other significant main and/or interaction effect indicates a similar synchronization to the flickering stimuli in both groups. Dimensional analyses within the CA group revealed no significant main or interaction effect regarding childhood adversity (general adversity exposure, threat, and neglect) and psychiatric symptoms (depression, anxiety, and psychosis).

| **Table S5** Linear mixed model to assess the effects of group on neural responses to the visual base stimulation in the oddball paradigm. | | | | |
| --- | --- | --- | --- | --- |
|  | **β** | **SE** | **95% CI** | ***p*** |
|  | | | | |
| Age | 0.01 | 0.04 | -0.06 – 0.08 | 0.757 |
| Sex | 0.21 | 0.15 | -0.10 – 0.52 | 0.181 |
| Group | 0.29 | 0.16 | -0.03 – 0.61 | 0.077 |
| Expression | 0.02 | 0.07 | -0.11 – 0.15 | 0.778 |
| ROI[LOT(P7,P9,PO7)]^a^ | -1.15 | 0.06 | -1.26 – -1.03 | **<0.001** |
| ROI[ROT(P8,P10,PO8)]^a^ | -0.71 | 0.06 | -0.82 – -0.60 | **<0.001** |
| Group * Expression | -0.08 | 0.09 | -0.26 – 0.11 | 0.407 |
| **Model_2_** | | | | |
| Age | 0.01 | 0.06 | -0.11 – 0.12 | 0.889 |
| Sex | 0.19 | 0.26 | -0.34 – 0.72 | 0.464 |
| Childhood adversity | -0.07 | 0.15 | -0.38 – 0.23 | 0.629 |
| Expression | -0.05 | 0.10 | -0.25 – -0.14 | 0.585 |
| Depression | 0.11 | 0.21 | -0.31 – 0.52 | 0.610 |
| Anxiety | 0.09 | 0.21 | -0.34 – 0.52 | 0.674 |
| Psychosis | 0.17 | 0.17 | -0.17 – 0.51 | 0.330 |
| Childhood adversity *Expression | 0.12 | 0.11 | -0.11 – -0.35 | 0.302 |
| Depression * Expression | 0.13 | 0.15 | -0.17 – 0.44 | 0.387 |
| Anxiety * Depression | -0.18 | 0.16 | -0.48 – 0.13 | 0.252 |
| Psychosis * Expression | -0.08 | 0.12 | -0.32 – 0.17 | 0.535 |
| **Model_3_** | | | | |
| Age | 0.02 | 0.06 | -0.10 – 0.13 | 0.781 |
| Sex | 0.29 | 0.26 | -0.23 – 0.81 | 0.272 |
| Threat | 0.24 | 0.14 | -0.03 – 0.52 | 0.081 |
| Expression | -0.05 | 0.10 | -0.25 – -0.14 | 0.588 |
| Neglect | 0.11 | 0.13 | -0.37 – 0.15 | 0.408 |
| Depression | 0.09 | 0.20 | -0.31 – 0.49 | 0.644 |
| Anxiety | 0.03 | 0.21 | -0.38 – 0.45 | 0.868 |
| Psychosis | 0.07 | 0.16 | -0.26 – 0.41 | 0.657 |
| Threat * Expression | 0.02 | 0.11 | -0.19 – -0.23 | 0.842 |
| Neglect * Expression | 0.06 | 0.10 | -0.14 – 0.26 | 0.549 |
| Depression * Expression | 0.15 | 0.15 | -0.15 – 0.46 | 0.324 |
| Anxiety * Expression | -0.15 | 0.15 | -0.46 – 0.15 | 0.329 |
| Psychosis * Expression | -0.06 | 0.13 | -0.30 – 0.19 | 0.652 |
| ^a^The reference level is ROI[MO(O1,O2,Iz,Oz)]. Post-hoc analyses further revealed larger responses at the ROT vs LOT region (β = 0.44, s.e. = 0.05, *p* < .0001). | | | | |

**Main analyses of the neural discriminative responses.** Full statistical results are reported in Table S6 and have been discussed in the main text.

| **Table S6** Linear mixed models to assess the effects of childhood adversity and symptoms on neural discriminative responses in the oddball paradigm. | | | | |
| --- | --- | --- | --- | --- |
|  | **β** | **SE** | **95% CI** | ***p*** |
| **Model_1_** | | | | |
| Age | -0.00 | 0.01 | -0.02 – 0.02 | 0.872 |
| Sex | -0.05 | 0.04 | -0.13 – 0.04 | 0.311 |
| ROI[LOT(P7,P9,PO7)] ^a^ | -0.03 | 0.03 | -0.09 – 0.02 | 0.209 |
| ROI[ROT(P8,P10,PO8)]^a^ | 0.03 | 0.03 | -0.03 – 0.08 | 0.343 |
| Group | 0.11 | 0.05 | 0.02 – 0.21 | **0.024** |
| Expression | -0.08 | 0.03 | -0.14 – -0.02 | **0.015** |
| Group * Expression | -0.17 | 0.05 | -0.26 – -0.08 | **<0.001** |
| **Model_2_** | | | | |
| Age | -0.01 | 0.02 | -0.04 – 0.03 | 0.657 |
| Sex | -0.06 | 0.08 | -0.21 – 0.10 | 0.465 |
| Childhood adversity | -0.04 | 0.05 | -0.13 – 0.06 | 0.439 |
| Expression | -0.25 | 0.03 | -0.32 – -0.19 | **<0.001** |
| Depression | -0.03 | 0.06 | -0.16 – 0.09 | 0.587 |
| Anxiety | 0.06 | 0.06 | -0.07 – 0.19 | 0.331 |
| Psychosis | 0.04 | 0.05 | -0.07 – 0.14 | 0.480 |
| Childhood adversity *Expression | -0.08 | 0.04 | -0.16 – -0.00 | **0.044** |
| Depression * Expression | 0.08 | 0.05 | -0.02 – 0.19 | 0.123 |
| Anxiety * Depression | -0.06 | 0.05 | -0.16 – 0.05 | 0.271 |
| Psychosis * Expression | 0.02 | 0.04 | -0.07 – 0.10 | 0.711 |
| **Model_3_** | | | | |
| Age | -0.01 | 0.02 | -0.04 – 0.03 | 0.702 |
| Sex | -0.05 | 0.08 | -0.21 – 0.11 | 0.524 |
| Threat | 0.04 | 0.05 | -0.06 – 0.15 | 0.408 |
| Expression | -0.25 | 0.03 | -0.32 – -0.19 | **<0.001** |
| Neglect | -0.08 | 0.05 | -0.17 – 0.02 | 0.104 |
| Depression | -0.04 | 0.06 | -0.17 – 0.08 | 0.491 |
| Anxiety | 0.05 | 0.07 | -0.08 – 0.19 | 0.415 |
| Psychosis | 0.03 | 0.05 | -0.08 – 0.13 | 0.574 |
| Threat * Expression | -0.15 | 0.04 | -0.24 – -0.07 | **0.001** |
| Neglect * Expression | 0.05 | 0.04 | -0.03 – 0.12 | 0.205 |
| Depression * Expression | 0.10 | 0.05 | -0.01 – 0.20 | 0.063 |
| Anxiety * Expression | -0.05 | 0.05 | -0.15 – 0.06 | 0.371 |
| Psychosis * Expression | 0.03 | 0.04 | -0.05 – 0.11 | 0.510 |
| ^a^The reference level is ROI[ MO(O1,O2,Iz,Oz)]. Post-hoc analyses further revealed no difference between responses at the LOT vs ROT region (β = -0.06, s.e. = 0.03, *p* = 0.07). | | | | |

**Supplementary analyses of the neural discriminative responses.** Since the CA group showed lower accuracy than the HC group during orthogonal task performance, we conducted supplementary analyses by fitting the same Model_1_ as in the main analyses but included orthogonal task accuracy as a covariate. The results indicated no significant effect of accuracy (β = 0.49, SE = 0.38, *p* = 0.202, 95% CI -0.26 – 1.25). Consistent with the main analyses, we observed a significant main effect of group (β = 0.12, SE = 0.05, *p* = 0.024, 95% CI 0.02 – 0.21), a significant main effect of expression (β = -0.08, SE = 0.03, *p* = 0.009, 95% CI -0.15 – 0.02), and a significant group by expression interaction effect (β = -0.16, SE = 0.05, *p* = 0.001, 95% CI -0.25 – -0.07).

**Supplementary analyses of reliability for the neural discriminative responses.** As expected, given the relatively noisy signals resulting of averaging only two runs, Pearson correlation results indicated moderate associations between responses to the two female sequences and the two male sequences, both for the angry condition (*r*(91) = 0.34, *p* < .001) and the happy condition (*r*(91) = 0.41, *p* < .001).

**Exploratory analyses of the neural discriminative responses.** Exploratory analyses were conducted to investigate the effect of individual differences in general CA exposure, early threat and neglect experiences, and each category of symptoms, while not controlling for each other, within the CA group. Model_supp1,_ exploring the impact of general CA exposure, revealed no significant effect (Table S7). Model_supp2,_ disentangling the effect of both adversity dimensions, revealed that the reduced neural sensitivity for angry faces and enhanced sensitivity for happy faces in participants with CA associated with individual differences in early threat experiences and not with individual differences in neglect experiences, which is in line with our main findings while controlling for the current symptoms. Model_supp3_, Model_supp4_ and Model_supp5_, exploring the effect of depressive, anxiety and psychotic symptoms, separately, while not controlling for adversity, showed no significantly modulating effect of symptoms, further confirming our main findings that individual variability in severity of symptomatology did not modulate the neural sensitivity for these facial expressions in our participant samples.

| **Table S7** Supplementary linear mixed models to assess the separate effect of childhood adversity and symptoms on neural discriminative responses in the oddball paradigm. | | | | |
| --- | --- | --- | --- | --- |
|  | **β** | **SE** | **95% CI** | ***p*** |
| **Model_supp1_** | | | | |
| Age | -0.01 | 0.02 | -0.04 – 0.02 | 0.459 |
| Sex | -0.03 | 0.07 | -0.18 – 0.12 | 0.673 |
| Childhood adversity | -0.01 | 0.04 | -0.09 – 0.07 | 0.825 |
| Expression | -0.25 | 0.03 | -0.32 – -0.18 | **<0.001** |
| Childhood adversity * Expression | -0.06 | 0.03 | -0.13 – 0.01 | 0.071 |
| **Model_supp2_** | | | | |
| Age | -0.01 | 0.02 | -0.04 – 0.02 | 0.563 |
| Sex | -0.02 | 0.08 | -0.18 – 0.13 | 0.749 |
| Threat | 0.06 | 0.04 | -0.03 – 0.15 | 0.182 |
| Expression | -0.25 | 0.03 | -0.32 – -0.19 | **<0.001** |
| Neglect | -0.07 | 0.04 | -0.17 – 0.02 | 0.103 |
| Threat * Expression | -0.11 | 0.04 | -0.19 – -0.04 | **0.003** |
| Neglect * Expression | 0.05 | 0.04 | -0.03 – 0.12 | 0.208 |
| **Model_supp3_** | | | | |
| Age | -0.01 | 0.02 | -0.04 – 0.02 | 0.497 |
| Sex | -0.05 | 0.07 | -0.20 – 0.10 | 0.533 |
| Depression | 0.02 | 0.04 | -0.06 – 0.10 | 0.682 |
| Expression | -0.25 | 0.03 | -0.32 – -0.18 | **<0.001** |
| Depression * Expression | 0.01 | 0.03 | -0.05 – 0.08 | 0.713 |
| **Model_supp4_** | | | | |
| Age | -0.01 | 0.02 | -0.05 – 0.02 | 0.439 |
| Sex | -0.06 | 0.08 | -0.21 – 0.10 | 0.446 |
| Anxiety | 0.04 | 0.04 | -0.04 – 0.12 | 0.309 |
| Expression | -0.25 | 0.03 | -0.32 – -0.18 | **<0.001** |
| Anxiety * Expression | -0.02 | 0.03 | -0.09 – 0.04 | 0.523 |
| **Model_supp5_** | | | | |
| Age | -0.01 | 0.02 | -0.04 – 0.02 | 0.595 |
| Sex | -0.05 | 0.08 | -0.20 – 0.10 | 0.482 |
| Psychosis | 0.03 | 0.04 | -0.05 – 0.11 | 0.419 |
| Expression | -0.25 | 0.03 | -0.32 – -0.18 | **<0.001** |
| Psychosis * Expression | -0.01 | 0.03 | -0.07 – 0.06 | 0.856 |

# Appendix S9: Supplementary results of the multi-input paradigm

**Orthogonal task performance.** For reaction times, results also revealed equal performances for the two groups and under the two categories. For the accuracy, results showed equal performances for the groups and under the two presentation rates.

| **Table S8** Linear mixed model to assess the orthogonal task performance of both groups in the multi-input paradigm. | | | | |
| --- | --- | --- | --- | --- |
|  | **β** | **SE** | **95% CI** | ***p*** |
| **Speed** | | | | |
| Age | -0.00 | 0.00 | -0.00 – 0.00 | 0.937 |
| Sex | -0.01 | 0.01 | -0.03 – 0.01 | 0.560 |
| Group | 0.01 | 0.01 | -0.01 – 0.03 | 0.430 |
| **Accuracy** | | | | |
| Age | -0.00 | 0.00 | -0.00 – 0.00 | 0.705 |
| Sex | 0.01 | 0.00 | -0.00 – 0.02 | 0.170 |
| Group | -0.00 | 0.00 | -0.01 – 0.01 | 0.689 |

**Main analyses of the neural responses.** Full statistical results are reported in Table S9 and have been discussed in the main text.

| **Table S9** Linear mixed models to assess the effects of childhood adversity and symptoms on neural responses in the multi-input paradigm. | | | | |
| --- | --- | --- | --- | --- |
|  | **β** | **SE** | **95% CI** | ***p*** |
| **Model_1_** | | | | |
| Age | -0.01 | 0.03 | -0.06 – 0.05 | 0.812 |
| Sex | 0.17 | 0.13 | -0.08 – 0.42 | 0.192 |
| Group | 0.19 | 0.13 | -0.07 – 0.45 | 0.156 |
| Expression | -0.26 | 0.06 | -0.39 – -0.14 | **<0.001** |
| Group * Expression | 0.20 | 0.09 | 0.02 – 0.37 | **0.026** |
| **Model_2_** | | | | |
| Age | -0.05 | 0.05 | -0.14 – 0.05 | 0.324 |
| Sex | 0.10 | 0.22 | -0.35 – 0.54 | 0.664 |
| Expression | -0.07 | 0.06 | -0.18 – 0.05 | 0.237 |
| Depression | -0.09 | 0.16 | -0.42 – 0.24 | 0.590 |
| Anxiety | 0.05 | 0.17 | -0.29 – 0.40 | 0.748 |
| Psychosis | 0.00 | 0.14 | -0.27 – 0.28 | 0.998 |
| Childhood adversity | 0.07 | 0.12 | -0.18 – 0.31 | 0.574 |
| Depression * Expression | 0.03 | 0.10 | -0.16 – 0.22 | 0.785 |
| Anxiety * Expression | 0.03 | 0.09 | -0.16 – 0.21 | 0.781 |
| Psychosis * Expression | 0.04 | 0.07 | -0.10 – 0.19 | 0.552 |
| Childhood adversity * Expression | 0.03 | 0.07 | -0.11 – 0.16 | 0.693 |
| **Model_3_** | | | | |
| Age | -0.03 | 0.05 | -0.13 – 0.06 | 0.471 |
| Sex | 0.15 | 0.22 | -0.28 – 0.59 | 0.477 |
| Expression | -0.07 | 0.06 | -0.18 – 0.05 | 0.237 |
| Depression | -0.11 | 0.16 | -0.43 – 0.21 | 0.498 |
| Anxiety | 0.01 | 0.17 | -0.32 – 0.35 | 0.941 |
| Psychosis | -0.02 | 0.13 | -0.29 – 0.25 | 0.873 |
| Threat | 0.26 | 0.13 | -0.02 – 0.53 | 0.065 |
| Neglect | -0.15 | 0.12 | -0.38 – 0.09 | 0.208 |
| Depression * Expression | 0.03 | 0.10 | -0.16 – 0.22 | 0.731 |
| Anxiety * Expression | 0.03 | 0.10 | -0.16 – 0.22 | 0.726 |
| Psychosis * Expression | 0.05 | 0.07 | -0.10 – 0.20 | 0.504 |
| Threat * Expression | -0.03 | 0.08 | -0.18 – 0.12 | 0.671 |
| Neglect * Expression | 0.06 | 0.07 | -0.07 – 0.19 | 0.382 |

**Supplementary analyses of the neural responses.** The same model_1_ as in the main analyses was fitted while only including the subjects who had eye-tracking data (28 HC and 39 CA). Results again revealed a group by expression interaction effect for the neural data (β = 0.24, SE = 0.09, *p* = 0.011, 95% CI 0.06 - 0.43). While controls showed higher neural responses to neutral relative to angry faces (β = 0.28, SE = 0.07, 95% CI 0.10 - 0.47), participants with adversity showed no difference in neural response to the two categories of faces (β = 0.04, SE = 0.06, 95% CI -0.12 - 0.20). In addition, the same tendency of larger responses to angry faces in the CA than in the HC group was also observed (β = 0.41, SE = 0.16, 95% CI -0.01 - 0.83).

**Supplementary analyses of reliability for the neural responses.** Statistical analyses revealed strong correlations between responses to neutral faces presented at 5 Hz and 6 Hz (four sequences each; *r*(85) = 0.67, *p* < .001). Similarly, responses to angry faces showed high correlation between 5 Hz and 6 Hz presentations (four sequences each; *r*(85) = 0.70, *p* < .001). Note that these correlations of the base rate responses are substantially larger as those for the oddball responses, as they have been estimated based on many more stimulus presentations per run.

**Exploratory analyses of the neural responses.** Exploratory analyses were conducted to investigate the effect of individual differences in general CA exposure (Model_supp1_), early threat and neglect experiences (Model_supp2_), and each category of symptoms (Model_supp3_, Model_supp4_ and Model_supp5_ with depressive, anxiety and psychotic symptoms as the predictor, separately), while not controlling for each other, on the neural responses within the CA group. In line with the main findings while controlling for each other, no significant effects were shown.

| **Table S10** Supplementary linear mixed models to assess the separate effect of childhood adversity and symptoms on neural responses in the multi-input paradigm. | | | | |
| --- | --- | --- | --- | --- |
|  | **β** | **SE** | **95% CI** | ***p*** |
| **Model_supp1_** | | | | |
| Age | -0.05 | 0.04 | -0.13 – 0.04 | 0.304 |
| Sex | 0.12 | 0.20 | -0.28 – 0.53 | 0.544 |
| Childhood adversity | 0.05 | 0.10 | -0.15 – 0.25 | 0.604 |
| Expression | -0.07 | 0.06 | -0.18 – 0.05 | 0.239 |
| Childhood adversity * Expression | 0.07 | 0.06 | -0.05 – 0.18 | 0.233 |
| **Model_supp2_** |  |  |  |  |
| Age | -0.03 | 0.04 | -0.12 – 0.06 | 0.476 |
| Sex | 0.16 | 0.20 | -0.24 – 0.55 | 0.434 |
| Expression | -0.07 | 0.06 | -0.18 – 0.05 | 0.242 |
| Threat | 0.20 | 0.11 | -0.03 – 0.42 | 0.083 |
| Neglect | -0.15 | 0.11 | -0.37 – 0.08 | 0.193 |
| Threat * Expression | 0.02 | 0.07 | -0.10 – 0.15 | 0.705 |
| Neglect * Expression | 0.06 | 0.07 | -0.07 – 0.19 | 0.381 |
| **Model_supp3_** |  |  |  |  |
| Age | -0.05 | 0.04 | -0.13 – 0.04 | 0.311 |
| Sex | 0.14 | 0.20 | -0.27 – 0.55 | 0.487 |
| Depression | -0.02 | 0.10 | -0.22 – 0.18 | 0.832 |
| Expression | -0.07 | 0.06 | -0.18 – 0.05 | 0.236 |
| Depression * Expression | 0.08 | 0.06 | -0.03 – 0.20 | 0.154 |
| **Model_supp4_** |  |  |  |  |
| Age | -0.05 | 0.04 | -0.13 – 0.04 | 0.301 |
| Sex | 0.12 | 0.21 | -0.30 – 0.54 | 0.571 |
| Anxiety | 0.01 | 0.10 | -0.19 – 0.22 | 0.895 |
| Expression | -0.07 | 0.06 | -0.18 – 0.05 | 0.235 |
| Anxiety * Expression | 0.08 | 0.06 | -0.03 – 0.20 | 0.159 |
| **Model_supp5_** |  |  |  |  |
| Age | -0.04 | 0.04 | -0.13 – 0.05 | 0.387 |
| Sex | 0.13 | 0.20 | -0.29 – 0.54 | 0.540 |
| Psychosis | 0.01 | 0.10 | -0.20 – 0.22 | 0.895 |
| Expression | -0.07 | 0.06 | -0.18 – 0.05 | 0.239 |
| Psychosis * Expression | 0.09 | 0.06 | -0.03 – 0.20 | 0.140 |

**Data quality of eye-tracking.** T-tests to compare the data quality of both groups demonstrated no group differences: both groups showed similar average error angles (mean_HC_ 0.01˚, s.d. = 0.003˚ vs mean_CA_ 0.01˚, s.d. = 0.003˚; *t*_(65)_ = 0.506, *p* = .669) and the root-mean-square of the angles (mean_HC_ 0.01, s.d. = 0.003 vs mean_CA_ 0.01, s.d. = 0.005; *t*_(65)_ = -0.01, *p* = .996).

**Main analyses of the visual looking patterns. Full statistical** results are reported in Table S11 and have been discussed in the main text.

| **Table S11** Linear mixed models to assess the effects of childhood adversity and symptoms on visual looking behaviours in the multi-input paradigm. | | | | |
| --- | --- | --- | --- | --- |
|  | **β** | **SE** | **95% CI** | ***p*** |
| **Model_1_** | | | | |
| Age | -0.01 | 0.01 | -0.02 – 0.01 | 0.392 |
| Sex | -0.00 | 0.03 | -0.06 – 0.06 | 0.993 |
| Group | -0.04 | 0.04 | -0.13 – 0.05 | 0.361 |
| Expression | -0.09 | 0.05 | -0.18 – -0.00 | **0.045** |
| Group * Expression | 0.06 | 0.06 | -0.06 – 0.18 | 0.302 |
| **Model_2_** | | | | |
| Age | -0.02 | 0.01 | -0.04 – -0.00 | **0.046** |
| Sex | 0.05 | 0.04 | -0.03 – 0.14 | 0.210 |
| Expression | -0.03 | 0.04 | -0.11 – 0.05 | 0.429 |
| Depression | 0.00 | 0.04 | -0.08 – 0.08 | 0.998 |
| Anxiety | -0.03 | 0.04 | -0.12 – 0.05 | 0.461 |
| Psychosis | -0.04 | 0.03 | -0.11 – 0.03 | 0.236 |
| Childhood adversity | 0.02 | 0.03 | -0.05 – 0.08 | 0.609 |
| Depression * Expression | 0.00 | 0.06 | -0.11 – 0.11 | 0.999 |
| Anxiety * Expression | 0.04 | 0.06 | -0.08 – 0.15 | 0.508 |
| Psychosis * Expression | 0.00 | 0.05 | -0.09 – 0.10 | 0.931 |
| Childhood adversity * Expression | -0.01 | 0.04 | -0.09 – 0.07 | 0.807 |
| **Model_3_** | | | | |
| Age | -0.02 | 0.01 | -0.04 – -0.00 | **0.045** |
| Sex | 0.05 | 0.04 | -0.04 – 0.14 | 0.246 |
| Expression | -0.03 | 0.04 | -0.11 – 0.05 | 0.432 |
| Depression | 0.00 | 0.04 | -0.08 – 0.09 | 0.976 |
| Anxiety | -0.03 | 0.04 | -0.11 – 0.06 | 0.500 |
| Psychosis | -0.04 | 0.03 | -0.11 – 0.03 | 0.259 |
| Threat | -0.00 | 0.04 | -0.08 – 0.07 | 0.903 |
| Neglect | 0.02 | 0.03 | -0.05 – 0.09 | 0.523 |
| Depression * Expression | -0.00 | 0.06 | -0.12 – 0.11 | 0.985 |
| Anxiety * Expression | 0.04 | 0.06 | -0.08 – 0.15 | 0.524 |
| Psychosis * Expression | 0.00 | 0.05 | -0.09 – 0.10 | 0.952 |
| Threat * Expression | 0.00 | 0.05 | -0.10 – 0.10 | 0.941 |
| Neglect * Expression | -0.01 | 0.05 | -0.10 – 0.08 | 0.748 |

**Exploratory analyses of the visual looking patterns.** Exploratory analyses of the separate effect of general CA exposure (Model_supp1_), early threat and neglect experiences (Model_supp2_), and each category of symptoms (Model_supp3_, Model_supp4_ and Model_supp5_ with depressive, anxiety and psychotic symptoms as the predictor, separately), while not controlling for each other, on the looking times within the CA group. In line with the main findings while controlling for each other, no significant effects were shown.

| **Table S12** Supplementary linear mixed models to assess the separate effect of childhood adversity and symptoms on visual looking behaviours in the multi-input paradigm. | | | | |
| --- | --- | --- | --- | --- |
|  | **β** | **SE** | **95% CI** | ***p*** |
| **Model_supp1_** | | | | |
| Age | -0.02 | 0.01 | -0.03 – 0.00 | 0.096 |
| Sex | 0.04 | 0.04 | -0.04 – 0.12 | 0.364 |
| Childhood adversity | -0.01 | 0.03 | -0.06 – 0.05 | 0.744 |
| Expression | -0.03 | 0.04 | -0.11 – 0.05 | 0.429 |
| Childhood adversity * Expression | 0.00 | 0.04 | -0.07 – 0.08 | 0.961 |
| **Model_supp2_** | | | | |
| Age | -0.02 | 0.01 | -0.03 – 0.00 | 0.079 |
| Sex | 0.03 | 0.04 | -0.05 – 0.11 | 0.428 |
| Expression | -0.03 | 0.04 | -0.11 – 0.05 | 0.430 |
| Threat | -0.03 | 0.03 | -0.10 – 0.03 | 0.316 |
| Neglect | 0.02 | 0.03 | -0.04 – 0.09 | 0.466 |
| Threat * Expression | 0.02 | 0.04 | -0.07 – 0.11 | 0.670 |
| Neglect * Expression | -0.02 | 0.04 | -0.11 – 0.07 | 0.691 |
| **Model_supp3_** | | | | |
| Age | -0.02 | 0.01 | -0.03 – 0.00 | 0.079 |
| Sex | 0.04 | 0.04 | -0.04 – 0.12 | 0.350 |
| Depression | -0.04 | 0.03 | -0.09 – 0.02 | 0.172 |
| Expression | -0.03 | 0.04 | -0.11 – 0.05 | 0.426 |
| Depression * Expression | 0.03 | 0.04 | -0.05 – 0.10 | 0.491 |
| **Model_supp4_** | | | | |
| Age | -0.01 | 0.01 | -0.03 – 0.00 | 0.111 |
| Sex | 0.05 | 0.04 | -0.03 – 0.13 | 0.245 |
| Anxiety | -0.05 | 0.03 | -0.10 – 0.01 | 0.092 |
| Expression | -0.03 | 0.04 | -0.11 – 0.04 | 0.424 |
| Anxiety * Expression | 0.04 | 0.04 | -0.04 – 0.11 | 0.334 |
| **Model_supp5_** | | | | |
| Age | -0.02 | 0.01 | -0.04 – -0.00 | **0.032** |
| Sex | 0.05 | 0.04 | -0.03 – 0.13 | 0.218 |
| Psychosis | -0.05 | 0.03 | -0.11 – 0.00 | 0.072 |
| Expression | -0.03 | 0.04 | -0.11 – 0.04 | 0.422 |
| Psychosis * Expression | 0.02 | 0.04 | -0.06 – 0.09 | 0.621 |

# **References**

Addolorato, G., Ancona, C., Capristo, E., et al. (1999). State and trait anxiety in women affected by allergic and vasomotor rhinitis. *Journal of Psychosomatic Research*, **46**, 283–89

Beck, A.T., Steer, R.A., Brown, G.K. (1996). *Manual for the Beck Depression Inventory-II.* San Antonio, TX: Psychological Corporation.

Bernstein, D.P., Fink, L. (1998). *Childhood Trauma Questionnaire: A retrospective self-report manual*. San Antonio: TX: The Psychological Corporation.

Croft, J., Heron, J., Teufel, C., et al. (2019). Association of Trauma Type, Age of Exposure, and Frequency in Childhood and Adolescence with Psychotic Experiences in Early Adulthood. *JAMA Psychiatry*, **76**, 79–86

Dennis, C.L., Coghlan, M., Vigod, S. (2013). Can we identify mothers at-risk for postpartum anxiety in the immediate postpartum period using the State-Trait Anxiety Inventory? *Journal of Affective Disorders*, **150**, 1217–20

Finkelhor, D., Hamby, S.L., Ormrod, R., et al. (2005). The Juvenile Victimization Questionnaire: Reliability, validity, and national norms. *Child Abuse & Neglect*, **29**, 383–412

Hessels, R.S., Niehorster, D.C., Kemner, C., et al. (2017). Noise-robust fixation detection in eye movement data: Identification by two-means clustering (I2MC). *Behavior Research Methods*, **49**, 1802–23

Langner, O., Dotsch, R., Bijlstra, G., et al. (2010). Presentation and validation of the Radboud Faces Database Presentation and validation of the Radboud Faces Database. *Cognition and Emotion*, **24**, 1377–88

Lenth, R., Singmann, H., Love, J., et al. (2022). Estimated marginal means, aka least-squares means

NICE Guidelines (2010). *Alcohol-use disorders: prevention*.

Pinheiro, J., Bates, D., & R Core Team. (2023). nlme: Linear and Nonlinear Mixed Effects Models. R package version 3.1-163. Retrieved from https://cran.r-project.org/package=nlme

R Core Team. (2022). R: A language and environment for statistical computing. R Foundation for Statistical Computing

Savill, M., D’Ambrosio, J., Cannon, T.D., et al. (2018). Psychosis risk screening in different populations using the Prodromal Questionnaire: A systematic review. *Early Intervention in Psychiatry*, **12**, 3–14

Spielberger, C.D., Gorsuch, R.L., Lushene, R., et al. (1983). *State-Trait Anxiety Inventory for Adults*. Consulting Psychologists Press.

Van der Donck, S., Dzhelyova, M., Vettori, S., et al. (2019). Fast Periodic Visual Stimulation EEG Reveals Reduced Neural Sensitivity to Fearful Faces in Children with Autism. *Journal of Autism and Developmental Disorders*, **49**, 4658–73

Van der Donck, S., Moerkerke, M., Dlhosova, T., et al. (2022). Monitoring the effect of oxytocin on the neural sensitivity to emotional faces via frequency‐tagging EEG: A double‐blind, cross‐over study. *Psychophysiology*, **59**, e14026

Van der Donck, S., Vettori, S., Dzhelyova, M., et al. (2021). Investigating automatic emotion processing in boys with autism via eye tracking and facial mimicry recordings. *Autism Research*, **14**, 1404–20

Vettori, S., Van der Donck, S., Nys, J., et al. (2020). Combined frequency-tagging EEG and eye-tracking measures provide no support for the “excess mouth/diminished eye attention” hypothesis in autism. *Molecular Autism*, **11**, 94

Vettori, S., Dzhelyova, M., Van der Donck, S., et al. (2020). Combined frequency-tagging EEG and eye tracking reveal reduced social bias in boys with autism spectrum disorder. *Cortex*, **125**, 135–48

Whisman, M.A., Richardson, E.D. (2015). Normative Data on the Beck Depression Inventory - Second Edition (BDI-II) in College Students. *Journal of Clinical Psychology*, **71**, 898–907
